# Poles Apart: Arctic and Antarctic *Octadecabacter* strains Share High Genome Plasticity and a New Type of Xanthorhodopsin

**DOI:** 10.1371/journal.pone.0063422

**Published:** 2013-05-06

**Authors:** John Vollmers, Sonja Voget, Sascha Dietrich, Kathleen Gollnow, Maike Smits, Katja Meyer, Thorsten Brinkhoff, Meinhard Simon, Rolf Daniel

**Affiliations:** 1 Department of Genomic and Applied Microbiology and Göttingen Genomics Laboratory, Institute of Microbiology and Genetics, Georg-August University of Göttingen, Göttingen, Germany; 2 Institute for Chemistry and Biology of the Marine Environment, University of Oldenburg, Oldenburg, Germany; Belgian Nuclear Research Centre SCK/CEN, Belgium

## Abstract

The genus *Octadecabacter* is a member of the ubiquitous marine *Roseobacter* clade. The two described species of this genus, *Octadecabacter arcticus* and *Octadecabacter antarcticus*, are psychrophilic and display a bipolar distribution. Here we provide the manually annotated and finished genome sequences of the type strains *O. arcticus* 238 and *O. antarcticus* 307, isolated from sea ice of the Arctic and Antarctic, respectively. Both genomes exhibit a high genome plasticity caused by an unusually high density and diversity of transposable elements. This could explain the discrepancy between the low genome synteny and high 16S rRNA gene sequence similarity between both strains. Numerous characteristic features were identified in the *Octadecabacter* genomes, which show indications of horizontal gene transfer and may represent specific adaptations to the habitats of the strains. These include a gene cluster encoding the synthesis and degradation of cyanophycin in *O. arcticus* 238, which is absent in *O. antarcticus* 307 and unique among the *Roseobacter* clade. Furthermore, genes representing a new subgroup of xanthorhodopsins as an adaptation to icy environments are present in both *Octadecabacter* strains. This new xanthorhodopsin subgroup differs from the previously characterized xanthorhodopsins of *Salinibacter ruber* and *Gloeobacter violaceus* in phylogeny, biogeography and the potential to bind 4-keto-carotenoids. Biochemical characterization of the *Octadecabacter* xanthorhodopsins revealed that they function as light-driven proton pumps.

## Introduction

The *Roseobacter* clade, a subclade of the *Rhodobacteraceae* belonging to the *Alphaproteobacteria*, is a phylogenetically coherent but physiologically and morphologically diverse group of predominantly marine bacteria [1]. The members of this clade comprise more than 38 genera and are present ubiquitously in marine habitats worldwide [2], [3]. Based on multi-locus sequence analysis (MLSA), this clade can be divided into at least 5 subclades [4], [5]. It has been suggested that horizontal gene transfer (HGT) has a large influence on the physiological heterogeneity and genomic diversity of this clade [5].

In sea ice microbial communities, *Roseobacters* are represented by the genus *Octadecabacter*. *O. antarcticus* constitutes up to 1% of the total bacterial community in the Southern Ocean, and *O. arcticus* up to 23% of the total bacterial community in Arctic sea ice [6]. These psychrophilic, heterotrophic and gas vacuole-containing bacteria were originally isolated from the lower 20 cm of annual sea ice of the Arctic and the Antarctic, respectively [7]. *O. arcticus* 238 and *O. antarcticus* 307 are the type strains of the genus *Octadecabacter* [8]. They are of particular interest for the comprehensive description of the *Roseobacter* clade, because of their bipolar distribution and unique sea ice habitat [9]. Sea ice is an extreme environment, which is characterized by strong gradients of salinity and temperature as well as low nutrient availability [10], [11]. Despite their geographical separation, both strains share >99% identity on 16S rRNA gene sequence level. However, DNA/DNA hybridization assays revealed an overall genome similarity of only 42% [8]. Since this value is well below the species threshold [12], these strains were classified as two separate species.

In a preliminary genome comparison, presence of rhodopsin genes affiliated to the xanthorhodopsin group [13], [14] was reported for the *Octadecabacter* strains [5]. Rhodopsins are highly diverse retinal-binding and photoactive membrane proteins [15], [16]. Bacteriorhodopsins, halorhodopsins and sensory rhodopsins function as light-driven proton pumps, chloride pumps, and signal transducers, respectively. They are predominantly of archaeal origin, but closely related to fungal rhodopsins [17]. Proteorhodopsins, however, are predominantly of bacterial origin and assumed to function as proton pumps [18]. Proton-pumping xanthorhodopsins were first described in *Salinibacter ruber* [13] and *Gloeobacter violaceus* [14]. The unique feature of the xanthorhodopsins of these organisms is binding of 4-keto-carotenoids as antenna pigments.

The aim of this study was a comprehensive genome analysis of the *Octadecabacter* type strains to elucidate relationships between these geographically separated type strains and identify genomic features linked to sea ice habitats. Ice-associated features include the xanthorhodopsin gene products of the *Octadecabacter* strains. These products represent a new subgroup of xanthorhodopsins that is functionally and phylogenetically distinct from previously described xanthorhodopsins.

## Materials and Methods

### Cultivation

*Octadecabacter* and *Gloeobacter* strains were obtained from the Centre de Ressources Biologiques de l'Institut Pasteur (CRBIP, Paris, France). A recombinant *Escherichia coli* clone harboring the subcloned proteorhodopsin gene of the environmental clone EBAC31A08 [19] was kindly provided by Edward F. DeLong (MIT, Cambridge, MA, USA). All strains used in this study are listed in Table 1.

**Table 1:** List of strains used in this study.

Strain	Description	Source
*Octadecabacter arcticus* 238	Type strain of *O. arcticus*	CRBIP, Paris, France
*Octadecabacter antarcticus* 307	Type strain of *O. antarcticus*	CRBIP, Paris, France
*Gloeobacter violaceus* PCC 7421	Type strain of *G. violaceus*	CRBIP, Paris, France
pBAD_EBAC31A08 in *E. coli* UT5600	Opsin gene of EBAC31A08 subcloned into expression vector pBAD in host *E. coli* UT5600	DeLong, E.F., MIT, Cambridge, MA, USA
pET24D_protRho2 in *E. coli* C43	Opsin insert of pBAD_EBAC31A08 subcloned into expression vector pET24D in host *E. coli* C43	This study
pET24D_oarRho2 in *E. coli* C43	Opsin gene of *O. arcticus 238* cloned in expression vector pET24D in host *E. coli* C43	This study
pET24D_oanRho2 in *E. coli* C43	Opsin gene of *O. antarcticus* cloned in expression vector pET24D in host *E. coli* C43	This study
pET24D_gviolRho2 in *E. coli* C43	Opsin gene of *G. violaceus* PCC 7421 in expression vector pET24D in host *E. coli* C43	This study
pET24D in *E. coli* C43	Expression vector pET24D in host *E. coli* C43	This study

*Octadecabacter* cells were grown in marine broth medium MB2216 [20] at 8°C. *Gloeobacter violaceus* cells were grown in BG-11 medium at 22°C. Recombinant *E. coli* cells were grown in LB medium supplemented with kanamycin (30 µg/mL) or ampicillin (100 µg/mL) at 37°C. All cultures were incubated under constant shaking.

### Sequencing and Annotation

The genomes of *O. arcticus* 238 and *O. antarcticus* 307 were sequenced using the Sanger approach (https://moore.jcvi.org/moore/). Gap closure and polishing were done using the Staden software package [21] and PCR-based techniques. Open reading frames (ORFs) were identified using YACOP [22] and GLIMMER [23], and manually corrected. Functional annotation was initially performed with the ERGO software tool [24] and manually corrected by comparison to the Swissprot, TrEMBL (http://kr.expasy.org/), and Interpro databases [25]. Genes associated with transposable elements (TEs), were classified via BLAST comparisons with the ISFinder database (http://www-is.biotoul.fr) [26].

The complete sequences of *O. arcticus* 238 and *O. antarcticus* 307 chromosomes and plasmids have been deposited in GenBank under accession numbers CP003742 (*O. arcticus* chromosome), CP003743 (pOAR118), CP003744 (pOAR160), CP003740 (*O. antarcticus* chromosome), and CP003741 (pOAN63).

### Determination of Orthologs

Orthologous protein sequences were identified by bidirectional best-hit analyses (BBH; often also referred to as reciprocal best-hit method, RBH) [27], [28] using BLAST (http://www.ncbi.nlm.nih.gov/). Only bidirectional best-hits with e-values lower than 1e-10 were considered. In order to filter and remove false hits based on short local alignments of conserved protein domains, sequence identities were determined by performing global alignments for each bidirectional best-hit using the Needleman-Wunsch algorithm [29]. As in previous comparative studies [5], [30], [31], a cutoff value of 30% sequence identity was chosen to identify orthologs. An additional cutoff value of 60% was used to determine the number of orthologs with highly conserved sequences, as the probability for equivalent functions is considerably higher at sequence identities >50% [32]. The *Octadecabacter* pan-genome was determined as the sum of all genes in both *Octadecabacter* strains.

### Genome Sequence Comparisons

Genome alignment-based synteny plots were done using the NUCmer tool of the MUMmer suite [33] implemented in the Integrated Microbial Genomes (IMG) system (http://img.jgi.doe.gov) [34]. Only genome sequences consisting of ten or less scaffolds were used to compare genome synteny. BLAST-based average nucleotide identities (ANIb) were determined using the JSpecies software (www.imedea.uib.es/jspecies/) [35].

### Phylogenetic Analyses

Genome sequences were obtained from the NCBI GenBank sequence database (www.ncbi.nlm.nih.gov) or from the J. Craig Venter Institute (http://www.jcvi.org). Reference protein and nucleotide sequences were obtained from the NCBI non-redundant (nr) database. For Multilocus Sequence Analysis (MLSA), protein-sequences of genes for which one ortholog but no paralog was found in every comparison strain were concatenated. Sequences were aligned using clustalW [36]. Neighbor-joining and maximum-likelihood trees were constructed using ARB v5.1 [37]. To calculate tree backbones only 16S sequences >1200 bp and complete rhodopsin protein sequences were used. Short partial sequences were added to the tree backbones using Parsimony. For phylogenetic analysis of 16S rRNA gene sequences a dataset was assembled, which contains type strains representing the *Roseobacter* clade. *Methylococcus capsulatus* ACM1292 and *Thiotrix nivea* JP2 were employed as outgroups. For the analyses of microbial rhodopsins and cyanophycin ligases, datasets containing representatives of all described subgroups were assembled (Supplementary Table S1 and Supplementary Table S2). For MLSA, a filter was employed to remove gapped positions prior to tree calculation.

### Screening of Metagenomic Databases

Several metagenomic datasets derived from freshwater, marine, hypersaline, thermophilic, and ice-associated habitats [38]–[49] (Table S3) available at the CAMERA (http://camera.calit2.net/) and MG-RAST (http://metagenomics.anl.gov/) databases were screened for rhodopsins via batched BLAST comparisons. Only samples from the surface region to 30 m depth were analyzed. To prevent bias caused by small survey sizes only metagenomes with more than 100 000 reads were evaluated. Query sequences were selected from a phylogenetic dataset based on rhodopsin sequences available at NCBI. To ensure sensitivity at least two representatives of each main group (proteorhodopsins, xanthorhodopsins, fungal rhodopsins, bacteriorhodopsins, halorhodopsins and sensory rhodopsins, Supplementary Table S1) were used as query sequences in primary tBLASTn analyses of the metagenome datasets using a non-stringent e-value cutoff of 1.

The resulting hits were verified and classified by secondary BLASTx comparisons using stringent e-value and alignment cutoffs. As short reads generally yield lower e-values than long reads, different e-value cutoffs depending on the average read length of the respective dataset were chosen. A cutoff of 1e-20 was used at average read lengths >150 bp and a cutoff of 1e-10 at average read length <150 bp. The resulting alignments had to cover at least 60% of the reference sequence or 80% of the query sequence. The NCBI RefSeq and the self-produced phylogenetic dataset were used as reference databases for verification and classification of the rhodopsin sequences. Total rhodopsin abundances were normalized against the total number of reads of the respective metagenome.

### Heterologous Expression of Rhodopsins

Rhodopsin gene sequences were amplified via PCR using rhodopsin-specific primers and the PCR extender system (5 PRIME Inc., Gaithersburg, USA) at annealing temperatures of 60°C according to the recommendations of the manufacturer. A poly-guanosine tail and NcoI and XhoI restriction sites were added to the 5′ ends of the primers to allow directed ligation into the expression vector. The resulting primer sequences were as follows (poly-guanosine tail and restriction sites are underlined): oanrho2f, (5′-GGGGGCCATGGAAACTTTATCACTGGTCAG-3′); oanrho2r, (5′-GGGGGCTCGAGTTACTCGGCGGGGACCGTCTTGGTGTTTTTGTCC-3′); oarrho2f, (5′-GGGGGCCATGGAAACATTATCATTGGGTCAATATG-3′); oarrho2r, (5′-GGGGGCTCGAGTTATTCAGCAGGGACTGCTGTCTTTATGGAATCGTTG-3′); gviolrho2f, (5′-GGGGGCCATGGGGATGTTGATGACCGTATTTTCTTCTGC-3′); gviolrho2r, (5′-GGGGCTCGAGCTAGGAGATAAGACTGCCTC CCGATTTATTTGC-3′); protrho2f, (5′-GGGGGGCCATGGATGAAATTATTACTGATATTAGGTAGTG TTATTGCACTTCCTACATTTGC-3′); and protrho2r, (5′-GGGGGCTCGAGTTAAGCATT AGAAGATTCTTTAACAGCAACATTCCA-3′). The resulting PCR products were cloned into the expression vector pET24D and subsequently used to transform *E. coli* Top10 cells as recommended by the manufacturer (Invitrogen, Carlsbad CA, USA). Fidelity of PCR products and constructs was verified by sequencing. For expression of the rhodopsin genes, *E. coli* C43 [DE3] (Lucigen Cooperation, Middleton WI, USA) was used as host. The resulting recombinant *E. coli* strains are listed in Table 1.

### Reconstitution of Rhodopsins with Chromophores

All-trans retinal was obtained from Sigma-Aldrich (Steinheim, Germany). Salinixanthin was extracted from *S. ruber* cultures according to Imasheva et al. [14] and Lutnaes et al. [50].

To isolate membrane fragments carrying heterologously produced rhodopsins, cells were lysed by sonication. Subsequently, cell debris was precipitated by centrifugation (3000 *g*, 8°C, 10 min) followed by precipitation of membrane fragments via ultracentrifugation of the supernatant (40,000 *g*, 8°C, 2 h). The membrane fragment-containing pellet was then suspended in 50 mM Tris buffer (pH 8) containing 5 mM MgCl_2_ [19]. The chromophores retinal and salinixanthin were stored as stock solutions in ethanol (10 mM and 1 mM, respectively) and were added to final concentrations of 10 µM. Absorbance spectra were recorded before and after addition of chromophores using a Lambda25 UV/Vis Spectrometer (PerkinElmer, Rodgau, Germany).

### Light-induced Proton Translocation

Proton-pumping function of heterologously expressed rhodopsins was detected directly in suspensions of host cells according to Beja et al. [19]. The cells were washed twice by centrifugation (5,000 *g*, 8°C, 10 min) with a non-buffered nutrient-free salt solution (10 mM NaCl, 10 mM MgSO_4_, 100 µM CaCl_2_). The nutrient-free cell suspensions were kept at room temperature for at least 15 minutes before measurement. Light-dependent proton translocation was detected via temporary fluxes in the acidification rate of the cell suspensions, using a WTW pH330i pH-meter and a Sentix81 pH electrode (WTW, Weilheim, Germany). A 500 W tungsten Halogen lamp was used as light source. The light was filtered through 15 cm of ice-cold water in order to block heat emitted from the light source. Additionally, the suspensions were kept at room temperature by partial submersion of the culture bottles in water. The temperature of the suspensions was monitored throughout the experiment.

## Results and Discussion

### General Genome Comparisons

The genome of *O. antarcticus* 307 consists of a 4.8 Mb chromosome and a 63 kb plasmid (pOAN63) whereas *O. arcticus* 238 harbors a 5.2 Mb chromosome and two plasmids of 118 kb and 160 kb (pOAR118 and pOAR160, respectively). The GC-content of both genomes is 55%, which is at the lower end of the typical GC-content of *Roseobacter* genomes (Table 2). The number of predicted protein-encoding genes is 4,683 in *O. arcticus* and 4,492 in *O. antarcticus*.

**Table 2:** General genome comparisons of *Roseobacter* clade members.

Number of genes							
Organism	GC- content	Genome Size [Mb]	Protein- coding	Pseudo- genes	RNA genes^a^	TEs/Mb	Sub- group^c^
*Phaeobacter gallaeciensis* 2.10	60%	4.16	3875	16	69	4	1
*Phaeobacter gallaeciensis* DSM17395	60%	4.23	3875	16	69	10	1
*Phaeobacter arcticus* DSM 23566	59%	5.05	4726	102	81	10	1
*Phaeobacter caeruleus* 13	63%	5.35	5146	81	108	13	1
*Phaeobacter daeponensis* DSM 23529	64%	4.64	4284	69	78	9	1
*Phaeobacter inhibens*T5	60%	4.13	3884	39	63	7	1
*Phaeobacter* sp. Y4I	64%	4.34	4132	1	69	13	1
*Nautella italica* R11	60%	3.82	3655	1	69	3	1
*Rhodobacterales* sp. MED193	57%	4.65	4535	0	70	15	1
*Rhodobacterales* sp. SK209-2-6	57%	4.56	4537	0	73	20	1
*Ruegeria* sp. TrichCH4B	59%	4.69	4734	1	79	20	1
*Ruegeria* sp. TM1040	60%	4.15	3864	6	94	7	1
*Ruegeria* lacuscaerulensis ITI-1157	63%	3.52	3608	3	63	14	1
*Ruegeria* sp. KLH11	58%	4.49	4269	5	64	24	1
*Ruegeria* sp. TW15	56%	4.49	4380	0	45	8	1
*Ruegeria* pomeroyi DSS-3	64%	4.60	4252	31	72	5	1
*Rhodobacterales* sp. R2A57	51%	4.14	4386	0	43	16	1
*Sulfitobacter* sp. NAS-14.1	60%	4.00	3962	0	64	23	2
*Sulfitobacter* sp. EE-36	60%	3.55	3474	0	68	11	2
*Sulfitobacter* sp. GAI101	59%	4.53	4202	1	55	15	2
*Oceanibulbus indolifex* HEL-45	60%	4.11	4153	0	55	16	2
*Roseobacter denitrificans* OCh 114	59%	4.33	4129	17	55	8	2
*Roseobacter litoralis* Och 149	57%	4.75	4537	0	40	14	2
*Rhodobacterales* sp. HTCC2083	53%	4.02	4177	2	47	32	2
*Citreicella* sp. SE45	67%	5.52	5425	2	72	18	3
*Citreicella* sp. 357	64%	4.60	4528	0	45	31	3
*Pelagibaca bermudensis* HTCC2601	66%	5.43	5452	0	62	23	3
*Sagittula stellata* E-37	65%	5.26	5067	0	54	17	3
*Oceanicola batsensis* HTCC2597	66%	4.44	4212	0	49	12	3
*Roseovarius* sp. 217	61%	4.76	4772	0	51	23	3
*Roseovarius* sp. TM1035	61%	4.21	4102	0	56	9	3
*Roseobacter* sp. AzwK-3b	62%	4.18	4145	0	52	37	3
*Roseovarius nubinhibens* ISM	64%	3.67	3547	0	58	2	3
*Loktanella* sp. CCS2	55%	3.50	3660	0	43	3	4
*Loktanella vestfoldensis* SKA53	60%	3.06	3068	0	49	9	4
*Loktanella* sp. SE62	62%	4.58	4596	0	43	8	4
***Octadecabacter antarcticus*** ** 307**	**55%**	**4.88**	**4492**	**361**	**48**	**74**	**4**
***Octadecabacter arcticus*** ** 238**	**55%**	**5.20**	**4683**	**411**	**49**	**175**	**4**
*Thalassiobium* sp. R2A62	55%	3.49	3696	0	48	34	4
*Oceanicola granulosus* HTCC2516	70%	4.04	3792	0	63	6	4
*Wenxinia marina* DSM 24838	71%	4.18	4045	0	59	10	4
*Ketogulonicigenium vulgare* Y25	62%	3.29	3213	0	74	5	4
*Ketogulonigenium vulgarum* WSH-001	62%	3.28	3054	0	71	6	4
*Dinoroseobacter shibae* DFL-12	66%	4.42	4186	33	52	22	5
*Jannaschias*p. CCS1	62%	4.40	4283	0	56	6	5
*Maritimibacter alkaliphilus* HTCC2654	64%	4.53	4712	0	48	9	-
*Rhodobacterales* sp. HTCC2150	49%	3.58	3667	0	46	20	-
*Rhodobacterales* sp. HTCC2255^b^	39%^b^	4.81^b^	4507^b^	0	86^b^	4^b^	-

TE, transposable element-associated genes.

a tRNA genes and rRNA genes.

b Genome sequence is contaminated with *E. coli* and may not be representative.

c Strains are sorted according to subclade affiliation as indicated by Newton et al. [5] and Supplementary Figure S1.

Bidirectional BLAST analyses showed that 76% of the genes in the *Octadecabacter* pan-genome have orthologs in at least one other *Roseobacter* clade member. These genes represent part of the shared *Roseobacter* pan-genome (core and flexible genome without singletons). The predicted gene products of most of these orthologs (85%) exhibit >60% sequence identity, indicating identical or equivalent function. The remaining orthologs may have adapted to new functions in the respective strains through mutation and genetic drift. Although the overall shared pan-genome comprises most of the *Octadecabacter* genes, only 32–50% of the *Octadecabacter* pan-genome is shared by individual *Roseobacter* strains. This reflects the overall diversity of the *Roseobacter* clade.

The two analyzed *Octadecabacter* strains shared approximately 2% of the genome content exclusively with each other. This indicates that on genomic level the genus *Octadecabacter* is less defined by unique features, but more by unique composition of shared features from the *Roseobacter* pan-genome. This module-like assembly of a *Roseobacter* genome is in accordance with previous observations. Characteristic phenotypical and selected genomic features occur in a “patchy” distribution along distantly related *Roseobacter* phylogenetic groups [2].

**Figure 1:**
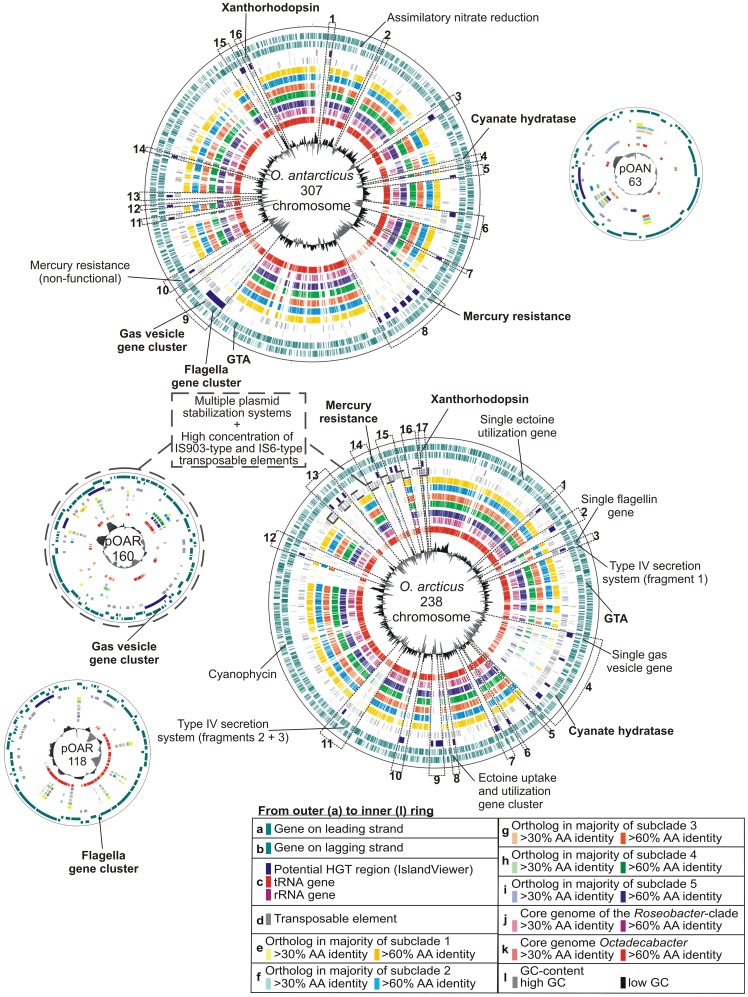
Circular representations of the *Octadecabacter* genomes. Regions of enhanced genome plasticity (RGP) are marked by dotted lines and numbered on the chromosomes of both strains. The location of protein-encoding genes, IslandViewer [49] predictions of potential genomic islands, rRNA genes, transposable elements (TEs) and orthologs to genes in strains of the different *Roseobacter* subclades as well as variations in GC-content are displayed. The comparison strains were grouped in subclades according to Newton et al. [5]. Several selected features indicative for horizontal gene transfer or intra-genomic recombination are labelled. Features that are present in both strains are marked in bold.

The unique genes of each strain comprise 20% of the genome in *O. antarcticus* and 23% in *O*. *arcticus*, indicating a high potential for individual adaptations. The majority of the unique and rare genes found in the *Octadecabacter* genomes are located in distinct regions of the chromosomes, thereby forming potential genomic islands (Figure 1) of which several were also indicated by IslandViewer [51] predictions. Regions containing multiple indicators for HGT such as IslandViewer predictions, divergent GC-content and low numbers of orthologs in closely related reference genomes were defined as “regions of enhanced genome plasticity” (RGP, Figure 1). These regions represent recombinatorial hot spots and were numbered Oar-RGP 1–17 on the chromosome of *O. arcticus* and Oan-RGP 1–16 on the chromosome of *O. antarcticus*. Plasmids were entirely defined as RGPs. Many of the below-described characteristic gene clusters are located in these regions (a general overview of RGP regions is provided in Supplementary Table S4).

### Phylogeny and Biogeography of the Genus *Octadecabacter*

**Figure 2:**
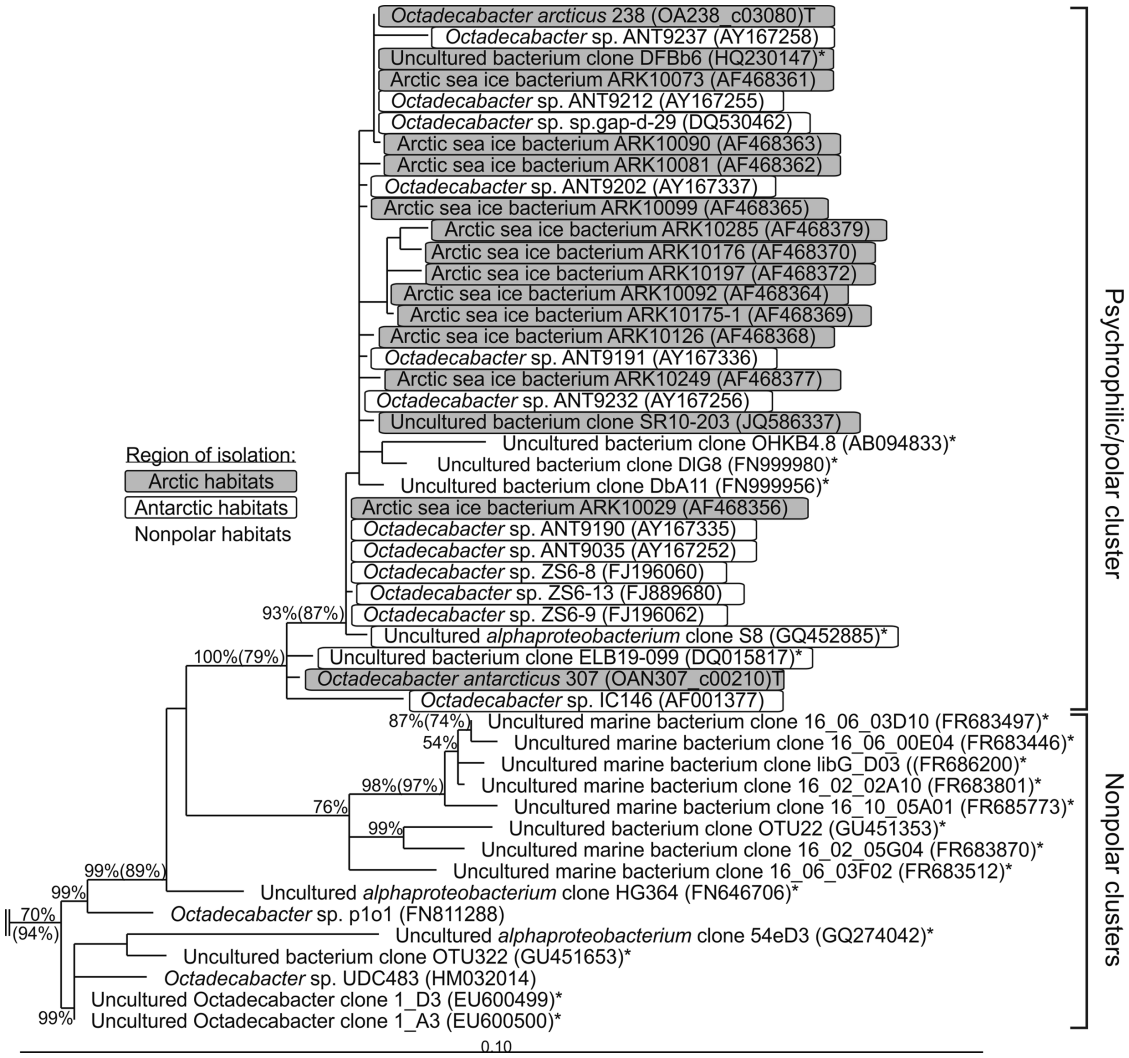
Phylogeny of *Octadecabacter* strains based on 16S rRNA gene sequences. Depicted is a subtree of a neighbor-joining tree of the complete *Roseobacter* clade, based on 16S rRNA gene sequences available at NCBI. The respective NCBI accession numbers are given in parentheses behind the individual clone or isolate designations. Sequences derived from clones are marked by an asterisk (*). Sequences derived from the type strain of a species are marked with a “T”. Neighbor-joining bootstrap values above 50% are given without parentheses at the respective nodes. For nodes that could be reproduced with maximum-likelihood calculation, the bootstrap values above 50% of the maximum-likelihood tree are given in parentheses. The 16S rRNA gene sequences of the type strains of *Methylococcus capsulatus* and *Thiothrix nivea* (NCBI-Accession-numbers AJ563935 and L40993, respectively) were used as outgroup (not shown).

Despite their close relationship on 16S rRNA gene sequence level, the genomes of *O. arcticus* 238 and *O. antarcticus* 307 exhibit significant differences in organization and content. This has been indicated by previously reported low DNA/DNA-hybridization values [8] and relatively high phylogenetic distances on MLSA level [5] (Supplementary Figure S1). The low resolution of 16S rRNA gene-based phylogeny with respect to closely related species has often been reported [52], [53]. Nevertheless, this approach has been proven to present reliable phylogenetic backbones, which are comparable to MLSA-based approaches [54], [55]. Thus, despite the differences of both *Octadecabacter* strains on genomic level, the 16S rRNA gene sequences indicate that Arctic and Antarctic strains are phylogenetically closely linked. Moreover, all *Octadecabacter* 16S rRNA gene sequences obtained from northern and southern polar habitats form a single distinct phylogenetic cluster (Figure 2). Only three sequences from non-polar habitats fall into this cluster, which originate from uncultivated organisms obtained from low temperature habitats: one from deep sea sediments (AB094833) and two from ciliates sampled in the Atlantic Ocean in winter (FN999980 + FN999956). The remaining non-polar sequences form separate clusters, indicating that psychrophilic strains of the opposite polar regions are more related to each other than to mesophilic strains from warmer regions between the two poles (Figure 2). Therefore, a direct link seems to exist between Arctic and Antarctic *Octadecabacter* populations.

### Genome Plasticity and Genetic Variability

**Figure 3:**
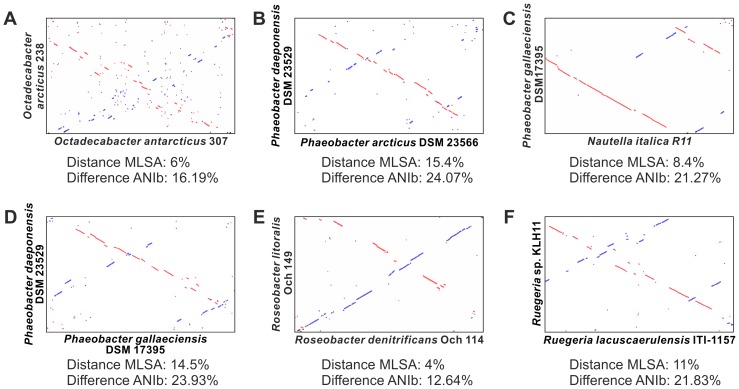
Synteny plots of the genomes of the *Octadecabacter* strains and other selected *Roseobacter* clade members. Synteny plots based on pairwise genome-alignments using MUMmer [33]. Linear regions indicate coherent regions of sequence homology. Homolog regions with identical orientation are displayed in red, inversions in blue. For reference the distance values obtained via multi locus sequence analysis (MLSA, Supplementary Figure S1) and the BLAST-based average nucleotide identities (ANIb) calculated using the JSpecies software [35] are given for each genome pair. Despite their close relationship on MLSA level and ANIb level, the *Octadecabacter* strains show a low genome synteny and many inversions (A). Other strains show much higher syntenies, despite similar oder more distant relationships on MLSA level and ANIb level (B–F). This includes strains isolated from globally opposite locations (C, D).

Whole-genome alignments of *O. arcticus* and *O. antarcticus* reveal only short continuous regions of sequence homology with a high frequency of inversions and strongly divergent organization along the genomes (Figure 3). This high divergence is in accordance with the low DNA/DNA-hybridization values of the *Octadecabacter* strains, which originally led to their classification as separate species [8]. However, it is not consistent with the genetic distances between these two organisms derived from MLSA-phylogeny or BLAST-based average nucleotide identity (ANIb). Other *Roseobacter* species with similar or higher genetic distances, including isolates from globally opposite locations [31], [56]–[58], exhibit much longer continuous stretches of sequence homology and less inversions in pairwise genome alignments (Figure 3). This implies that rearrangements occur more frequently in the genomes of the *Octadecabacter* strains than in other *Roseobacters*. Indications for intra-genomic rearrangements were mainly found in the genome of *O. arcticus* 238, which contained the most transposable elements of both *Octadecabacter* strains. Fragments of several gene clusters are scattered across multiple RGPs and even multiple replicons in this genome. Examples include the fragmentation of the type IV secretion system into three partial clusters found in Oar-RGP 3 and 11, and the presence of single flagella and gas vesicle genes in Oar-RGP 3 and 4 in addition to the complete gene clusters on plasmids pOAR118 and pOAR160 (Figure 1). Furthermore, an exchange of genetic material between the plasmid pOAR160 and the chromosomal area between Oar-RGP 13 and 17 is indicated by several shared features such as related plasmid stabilization systems and high concentrations of certain IS elements (Figure 1). The high frequency of recombination events is probably caused by the unusually large number and high diversity of transposable elements (TEs) such as IS elements or transposons in both *Octadecabacter* genomes (Tables 2 and 3). Multiple copies of TEs in a genome are known to indirectly facilitate inversion, deletion and translocation of large genomic areas via homologous recombination events [59], [60]. Accordingly, a higher number of TEs should result in a higher probability of recombination events. Based on the number of TE-associated mobility genes per megabase, the average density of these genes in all sequenced *Roseobacter* genomes is approximately 21 per megabase, whereas it is 4–8 times higher in the genomes of both *Octadecabacter* strains (Table 2). The TE-associated genes of *O. arcticus* and *O. antarcticus* fall into at least 21 and 16 families of IS elements, respectively (Table 3). The families IS3 and IS5 can be further divided into distinct subgroups [61], of which several are represented in the *Octadecabacter genomes*. This high diversity of IS elements in the *Octadecabacter* genomes is indicative for multiple independent HGT events from different sources. By acting as recombinational anchors for illegitimate recombination events [62], [63], TEs can also enhance the probability of HGT [64]. An enhanced potential for incorporation of new genetic material would explain the large number and size of RGPs in the *Octadecabacter* genomes. This might be of relevance in sea ice microbial communities, as Collins and Demming have described sea ice as rich in extracellular DNA and as a potential hot spot for HGT [65], [66]. Type IV secretion systems are known to be involved in transfer and uptake of DNA [67], but in *O. arcticus* the corresponding gene cluster is fragmented due to intra-genomic recombination (Figure 1 and Supplementary Figure S2A). New genomic features can be efficiently spread throughout *Octadecabacter* populations via gene transfer agents (GTAs). GTAs are phage-like particles that package and transfer random fragments of the host genome. GTAs are conserved in most members of the *Roseobacter* clade, including both *Octadecabacter* strains [5]. The function of GTAs as a mechanism for HGT between closely related *Roseobacter* strains has been demonstrated for *Silicibacter pomeroyi* DSS-3 [68].

**Table 3:** Families of transposable elements in the *Octadecabacter* strains.

	*O. arcticus* 238			*O. antarcticus* 307		
Family	Genes^a^	Pseudo-genes	Total^b^	Genes^a^	Pseudo-genes	Total^b^
IS3^c^	177	30	207	137	49	186
IS4	0	0	0	1	1	2
IS5^d^	44	19	63	34	8	42
IS6	25	4	29	12	3	15
IS10	7	8	15	0	0	0
IS21	11	0	11	13	4	17
IS30	45	17	62	8	0	8
IS66	66	15	81	2	1	3
IS91	22	5	27	12	0	12
IS110	17	4	21	33	39	72
IS200/IS605	38	2	40	14	0	14
IS204/IS1001/IS1096/IS1165	0	2	2	0	0	0
IS256	72	21	93	9	3	12
IS481	33	3	36	1	16	17
IS630	73	10	83	2	3	5
IS116/IS110/IS902	1	0	1	0	0	0
IS1182	0	0	0	1	14	15
IS1380	1	0	1	2	0	2
IS1595	33	1	34	20	3	23
ISAs1	28	2	30	0	0	0
ISL3	29	10	39	0	0	0
P4-integrase	1	0	1	0	0	0
ISNCY	1	2	3	0	0	0
unclassified	188	108	296	60	121	181
Total TEs	912	263	1175	361	265	626

a Protein-coding genes.

b Protein-coding genes and pseudogenes.

c Represented subgroups: IS3, IS51, IS407, IS150 [61].

d Represented subgroups: IS5, IS427, IS903, IS1031 [61].

High genome plasticity mediated by TEs could explain the divergence in genome content and genome organization of both *Octadecabacter* strains. The strong discrepancy between phylogenetic distances obtained by 16S rRNA gene-based (Figure 2) and MLSA-based approaches (Supplementary Figure S1) may result from an enhanced mutagenic activity. The 16S rRNA gene sequence exhibits a significantly lower rate of evolutionary substitution than protein-encoding genes [52], [69]. Consequently, evolutionary changes manifest earlier and more significantly in protein-encoding genes. In the *Octadecabacter* genomes, a high mutagenicity is indicated by an unusually large number of pseudogenes (411 in *O. arcticus* and 361 in *O. antarcticus*; Table 2). Most of these pseudogenes are TE-associated or carry an insertion of a TE (339 in *O. arcticus* and 282 in *O. antarcticus*). However, approximately 20% of the pseudogenes in both strains are not directly linked to TEs and comprise approximately 1.5% of the total genes in each *Octadecabacter* strain. This degree is above average for members of the *Roseobacter* clade (Table 2). Thus, polar *Octadecabacter* populations seem to be subjected to mutagenic influences other than TEs.

### Characteristic Features of the *Octadecabacter* Strains

The *Octadecabacter* strains are characterized by several features that are rare or even unique among the *Roseobacter* clade. Many of these show indications of HGT, such as location in a region of enhanced genome plasticity (RGP), flanking transposases and sequence similarities to organisms of different taxa. The Arctic and the Antarctic strain are distinguished by the presence of a gene cluster encoding synthesis and degradation of cyanophycin, which is only present in *O. arcticus* (Supplementary Figure S2B). Cyanophycin is a non-ribosomally synthesized branched polypeptide [70], which functions as a nitrogen storage compound in diverse bacteria [71], [72]. Ten phylogenetically distinct groups of cyanophycin ligases (Groups I–X) were established by Füser and Steinbüchel [71] based on protein sequences that were publicly available in 2007. Due to the increase of publicly available protein sequences at least 9 additional groups can be distinguished today (Groups XI–XIX, Supplementary Figure S3), but the groups previously described by Füser and Steinbüchel remain valid. The cyanophycin ligase of *O. arcticus* is affiliated with group IV [71], which consists of 31 mostly gammaproteobacterial sequences (Supplementary Figure S3). The *Octadecabacter* ligase is most closely related to the cyanophycin ligase of *Colwellia psychrerythraea* 34H, which is also an Arctic sea ice bacterium [73], [74]. Diverse cyanophycin ligases, including members of group VI, were found in Antarctic marine metagenomes. Therefore the absence of this trait in *O. antarcticus* cannot be directly attributed to differences in northern and southern polar habitats. Although this feature is not located within a RGP region in *O. arcticus*, its relationship to mostly gammaproteobacterial cyanophycin ligases indicates an origin via HGT. Instead of a cyanophycin pathway, *O. antarcticus* possesses genes for assimilatory nitrate and nitrite reduction pathways (Supplementary Figure S2C), which are present in 20 other *Roseobacter* clade members but not in *O. arcticus*. Like the cyanophycin genes in *O. arcticus*, these genes are also not located within an RGP. However, in *O. antarcticus* the corresponding gene cluster is closely flanked by TEs. The distribution of this feature among *Roseobacter* clade members is not linked with phylogeny [5], indicating that this feature may be frequently transferred via HGT. Another feature involved in nitrate metabolism are genes encoding cyanate hydratases. Cyanate is a toxic substance, which can accumulate in organisms as a byproduct of metabolic pathways such as the urea cycle [75], [76]. Cyanate hydratases allow the detoxification of cyanate as well as its utilization as a nitrogen source [77]. Corresponding gene clusters can be found in both *Octadecabacter* chromosomes (Oar-RGP 4 and Oan-RGP 4, Supplementary Figure S4A) but are absent in most other members of the *Roseobacter* clade.

Mercury resistance gene clusters [78] are also present in both *Octadecabacter* chromosomes (Supplementary Figure S4B). *O. arcticus* has one copy of this gene cluster in region Oar-RGP 14 whereas *O. antarcticus* possesses two copies, one without mutations in Oan-RGP 8 and one with a frameshift mutation in *merT* in Oan-RGP 10. Bidirectional BLAST analyses revealed similar gene clusters only in 10 of the 46 *Roseobacter* clade reference organisms.

A gene cluster encoding the formation of gas vesicles, a main characteristic of polar *Octadecabacter* strains, is located in Oan-RGP 9 of the *O. antarcticus* chromosome and the *O. arcticus* plasmid pOAR160 (Supplementary Figure S4C). Homologs of all 8 genes (*gvpAJM*, *gvpLF*, *gvpG*, *gvpO* and *gvpK)* that are essential for gas vesicle formation in *Halobacterium salinarum* PHH1 [79] were found. In addition, the *Octadecabacter* gene cluster contains genes encoding the chaperone GvpN and a conserved protein of unknown function. Heterotrophic bacteria containing gas vesicles are not common in marine habitats, except for polar sea ice microbial communities [7] in which they may function as dispersal mechanisms [9]. Gas vesicle genes can also be found in two *Loktanella* strains. *L.* sp. CCS2 harbors a *gvpK* gene but no complete gene cluster, whereas *L.* sp. SE-62 contains several gas vesicle genes in a coherent gene cluster. BLAST comparisons showed that these genes show low similarities to the *Octadecabacter* gas vesicle genes and are closer related to a gene cluster of *Rhodobacter capsulatus* SB1003. Furthermore, the corresponding gene clusters are located close to a photoactive yellow protein in *L.* sp. SE-62 as well as in *R. capsulatus* SB1003 [80]. This indicates that gas vesicles of the *Loktanella* strains are not directly related to those of the *Octadecabacter* strains but may share the same phylogenetic history as the gas vesicles of *R. capsulatus*.

The genomes of both *Octadecabacter* strains contain three large gene clusters encoding flagella synthesis (Supplementary Figure S4D), which show only low similarities to the majority of *Roseobacter* flagella gene clusters. The flagella gene clusters are organized almost identically in both *Octadecabacter* strains, although they are located in different replicons. In *O. arcticus* they are located on plasmid pOAR118 whereas in *O. antarcticus* they are present in Oan-RGP 9 of the chromosome. The *Octadecabacter* strains have been described as non-motile [8] and showed no swimming or swarming under various conditions (data not shown). Thus, the function of the flagella genes remains to be elucidated. Similar flagella gene clusters were only found in *L. vestfoldensis* SKA53, *Rhodobacterales* bacterium HTCC2083, and *Roseovarius* sp. TM1035.

A gene cluster encoding an ectoine uptake and utilization pathway is present in region Oar-RGP 8 of *O. arcticus* (Supplementary Figure S2D). Ectoine is a compatible solute involved in osmoprotection [81], and might be of importance for survival in the hypersaline brine channels of sea ice. The ectoine gene cluster is flanked by IS3 family TEs and consists of the ABC-transporter genes *ehuABCD* and the ectoine utilization genes *eutABCDE*. A paralog of *eutD* also flanked by IS3 family elements can be found near Oar-RGP 1 (Figure 1), indicating a TE mediated intra-genomic recombination event.

### A Novel Xanthorhodopsin Subgroup

**Figure 4:**
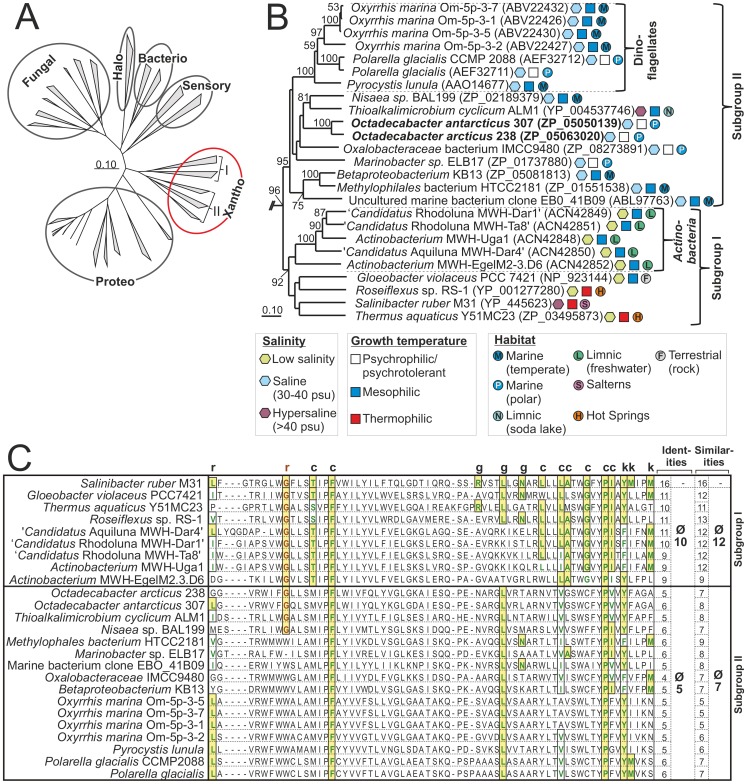
Comparison of the *Octadecabacter* rhodopsins with other microbial rhodopsins. (A) Unrooted neighbor-joining tree based on the amino acid sequence of representative members of all known groups of microbial rhodopsins (fungal, sensory, halo-, bacterio-, proteo-, and xanthorhodopsins). The two subgroups of xanthorhodopsins are indicated by the roman letters I and II. (B) Rooted detailed view of the xanthorhodopsin-branch of the same tree. Fungal rhodopsins served as outgroup. Bootstrap-values >50 are given at the respective nodes. The subgroups I and II as well as the *Actinobacteria* and Dinoflagellate subclusters are indicated by brackets. The lifestyle of the associated organisms is indicated by different symbols. For each rhodopsin the NCBI accession number is given in parentheses. (C) Alignment of the putative keto-carotenoid-binding region of xanthorhodopsins. Residues that interact with the keto-carotenoid in *Salinibacter* xanthorhodopsin as identified by Imasheva et al. [14] are marked by the letters c, g, k and r, which indicate contact with the chain, glucoside, keto group and ring of the carotenoid, respectively. Similarities are marked by solid boxes and colored letters. Identities are additionally marked by background shading. Amino acids corresponding to Gly_156_ of *Salinibacter* xanthorhodopsin are highlighted in red. The number of identities and similarities are given for each xanthorhodopsin and as an average for each subgroup (excluding *Salinibacter* xanthorhodopsin).

Gene clusters encoding a rhodopsin affiliated to the xanthorhodopsin group [14] were identified on the chromosomes of *O. arcticus* and *O. antarcticus* [5]. Several rhodopsin sequences available at the NCBI database are affiliated to this phylogenetically distinct and coherent group (Figure 4A). The *Rhodobacteraceae* strain HTCC2255 also possesses a rhodopsin gene cluster [5], however this cluster is affiliated to the proteorhodopsin group and not directly related to the *Octadecabacter* xanthorhodopsins. Based on sequence similarities this group can be divided in two main subgroups, here designated subgroup I and subgroup II (Figure 4A and B). This classification is supported by high bootstrap values of 95% (subgroup I) and 92% (subgroup II) and robust Hidden-Markov models (data not shown). Such a differentiation of xanthorhodopsins has not been previously reported. Both of the functionally characterized xanthorhodopsins, the rhodopsins of *Salinibacter ruber* and *Gloeobacter violaceus* fall into subgroup I. This subgroup also includes a noteworthy cluster of rhodopsins, designated actinorhodopsins, which is found exclusively in *Actinobacteria* [82]. The xanthorhodopsins of *O. arcticus* and *O. antarcticus* are affiliated to subgroup II and the first members of subgroup II that are functionally described (see below). Also included in this subgroup is a cluster of rhodopsins found in marine eukaryotes [83].

One difference between members of subgroup I and subgroup II is the organization of the corresponding gene clusters. All subgroup II xanthorhodopsins are organized in conserved gene clusters (Supplementary Figure S5), which are similar to the majority of proteorhodopsin gene clusters. These gene clusters consist of an opsin-encoding gene and several genes for retinal synthesis [15], [84], [85]. The gene clusters of subgroup I xanthorhodopsins are highly divergent and lack orthologs to conserved retinal synthesis genes found in subgroup II xanthorhodopsin and proteorhodopsin gene clusters. Several retinal synthesis genes have been identified in separate locations of the *S. ruber* genome [86], but these genes were more similar to the corresponding genes of halophilic archaea than to genes of other rhodopsin-harboring bacteria [15], [86]. Based on bidirectional best-hit analyses, most of these genes were not orthologous to retinal synthesis genes of subgroup II xanthorhodopsin-harboring organisms. Correspondingly, no orthologs of the β-carotene oxygenase gene *crtO,* which is associated with the subgroup I xanthorhodopsins of *S. ruber* [86] and *T. aquaticus* (Supplementary Figure S5), were identified in subgroup II xanthorhodopsin-harboring organisms.

### Ecology of Xanthorodopsin Subgroups

**Figure 5:**
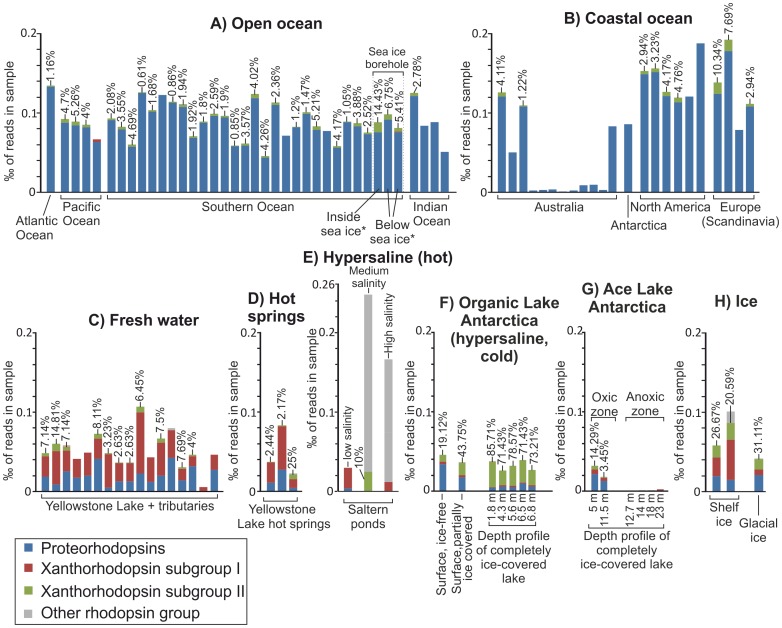
Abundance and diversity of rhodopsins in 454 sequencing-based metagenomes. The height of each bar indicates the normalized total abundance of rhodopsins, given in ? of reads in the respective metagenomic sample. The relative abundances of different rhodopsin groups are indicated by the relative color proportions in each bar. The relative abundances of subgroup II xanthorhodopsins are given in percent above the respective bars. The results for Sanger sequencing-based metagenomes are shown in a separate figure (Supplementary Figure S6). The represented metagenomes are listed in Supplementary Table S3.

Like most rhodopsins, closely related xanthorhodopsins can be found in diverse bacterial taxa [14], [15], indicating that this feature is often transmitted via HGT. Almost all subgroup II xanthorhodopsin sequences available from the NCBI nr database were obtained from mesophilic to psychrophilic marine microorganisms (Figure 4B). The only exception is the xanthorhodopsin from *Thioalkalimicrobium cyclicum* ALM1, isolated from a limnic hypersaline environment (Mono Lake, California, USA) [87]. More than a third of the subgroup II xanthorhodopsins (six sequences) was found in psychrophilic organisms from polar regions. In contrast, all subgroup I xanthorhodopsin sequences originate from mesophilic to thermophilic organisms derived from non-marine environments. The xanthorhodopsins of the actinorhodopsin cluster were obtained from freshwater habitats [82] whereas *Gloeobacter violaceus* was isolated from limestone rock [88]. The remaining three subgroup I members originate from thermotolerant [89] and thermophilic organisms [90] that have been isolated from saltern ponds and hot springs, respectively. This indicates that the xanthorhodopsin subgroups differ in their habitat distribution. To test this assumption several metagenomes from various environments were analyzed for the relative abundances of the different rhodopsin types (Figure 5 and Supplementary Figure S6).

The most abundant rhodopsin type in all marine metagenome samples was proteorhodopsin. Xanthorhodopsin sequences of both subgroups were present but only in low abundances. On average they constitute approximately 1–2% of the total rhodopsin sequences in marine environments. In 454 sequencing-based marine metagenomes xanthorhodopsins were mainly represented by subgroup II (Figure 5A and B), whereas in Sanger sequencing-based marine metagenomes they were more often represented by subgroup I (Supplementary Figure 6A and B). Both xanthorhodopsin subgroups were generally more abundant in freshwater, hot springs, and hypersaline habitats than in marine environments. This indicates that none of the subgroups is a characteristic trait of marine microorganisms. The fact that almost all subgroup II xanthorhodopsins originate from marine isolates (Figure 4) may be due to cultivation bias.

Subgroup I members were on average the most abundant rhodopsin type in freshwater and hot spring habitats, and comprise more than 50% of the total rhodopsin sequences in most samples (Figure 5C and D, Supplementary Figure S6C and D). Subgroup II xanthorhodopsins were also present in these samples and comprised 0–14% of the total rhodopsin sequences in freshwater habitats and 0–25% in hot springs. In warm hypersaline habitats such as solar salterns or tropical hypersaline lagoons xanthorhodopsins constituted varying but substantial fractions of the total rhodopsins. These fractions were dominated by subgroup I in three of the four analyzed hypersaline samples (Figure 5E, Supplementary Figure S6E).

Xanthorhodopsins of both subgroups were identified as the major type of rhodopsin in icy environments such as glacial ice and shelf ice (Figure 5H). This was also the case for subgroup II xanthorhodopsins in Organic Lake, Antarctica. Organic Lake is a shallow, eutrophic, and hypersaline lake with extremely low water temperatures [91], [92]. The relative abundance of subgroup II xanthorhodopsins was lowest (approximately 19%) when the lake was free of ice, increased (approximately 44%) when it was partially covered by ice, and highest (>70%) when it was completely covered by ice (Figure 5F). This shows that xanthorhodopsins, especially those of subgroup II, are mostly associated with psychrophilic organisms, indicating a possible evolutionary advantage over other rhodopsins in icy environments. A depth profile of the marine-derived Ace Lake (Antarctica) during complete ice coverage [38] yielded lower relative xanthorhodopsin abundances than Organic Lake (3–0% at depths >10 m). However, the uppermost sample (5 m depth) of Ace Lake still showed a higher relative abundance (approximately 14%) of subgroup II than any non-ice associated marine sample (Figure 5G). The generally lower rhodopsin abundances compared to Organic Lake may be due to the larger depth-range of the samples in Ace Lake (5–24 m compared to 0–7 m), but also to several factors that differentiate these two environments such as water temperature and salinity [91], [93]. Nonetheless, the importance of subgroup II xanthorhodopsins in icy environments is supported by the fact that the relative abundance of this subgroup was higher (approximately 14%) in seawater sampled directly from inside a borehole drilled through a 3 m thick sheet of pack ice than in the underlying water body (5–7%) or in any other marine sample (0–10%, Figure 5A).

### Functional Characterization of Xanthorhodopsin Subgroups

The *Octadecabacter* opsins were heterologously expressed in *E. coli* cells. Subsequently, the resulting recombinant *E. coli* strains displayed a characteristic pink color after addition of retinal (data not shown). Spectral analysis of membrane fragment suspensions revealed absorption maxima at 533(±1) nm and 535(±1) nm for the xanthorhodopsins of *O. arcticus and O. antarcticus*, respectively (Supplementary Figure S7). These values are higher than the absorption maximum of the reference proteorhodopsin from EBAC31A08 (521±1 nm), but lower than those of the subgroup I xanthorhodopsins from *Gloeobacter* (540±1 nm) and *Salinibacter* (560 nm) [94].

The rhodopsins were also analyzed for their potential to bind keto-carotenoids. Based on the crystal structure of *Salinibacter* xanthorhodopsin [95], Imasheva et al. [14] identified 16 amino acid residues that form the keto-carotenoid binding site and are positioned around the E and F helices of the protein. *Gloeobacter* xanthorhodopsin, which shares the ability to bind 4-keto-carotenoids [14], [96], harbors 11 identical amino acids at the corresponding positions (Figure 4C). This indicates that not all of the residues predicted in *Salinibacter* xanthorhodopsin are essential for this function. The residues forming this keto-carotenoid-binding site are conserved among subgroup I. On average 10 identical amino acids were located in the corresponding positions, whereas subgroup II xanthorhodopsins show only five correlating amino acid residues (Figure 4C). The functionally most important difference between subgroup I and subgroup II proteins is the amino acid residue in position 156 with respect to *Salinibacter* xanthorhodopsin [96]. All subgroup I members contain a glycine residue at this position whereas the majority of subgroup II harbors a tryptophan. It has been shown for the subgroup I xanthorhodopsins of *Salinibacter* and *Gloeobacter* that this residue is part of the keto-ring binding pocket. Due to its relatively small size, a glycine at this position provides space for a keto-ring, but substitution with a bulky tryptophan abolishes binding of keto-carotenoids [14]. Thus, most of the xanthorhodopsins belonging to subgroup II lack this requirement for keto-carotenoid binding, except the proteins from *O. arcticus*, *O. antarcticus*, *T. cyclicum* and *Nisaea* sp. BAL199. However, in all subgroup II xanthorhodopsins differences also occur at positions corresponding to Thr160, Leu194, Leu197, Ala198, Gly201 and Ile205 of *Salinibacter* xanthorhodopsin. These positions are part of a binding slot along helix F, which harbors the polyene chain of the keto-carotenoid. The absence of this slot in all subgroup II xanthorhodopsins affects keto-carotenoid-binding. This is supported by analysis of difference spectra after addition of crude salinixanthin extracts to membrane fragments harboring heterologously expressed xanthorhodopsins (Supplementary Figure S7). Vibrionic bands at 456, 480 and 521 nm that are typical for binding of salinixanthin [14], [96] were observed in *Gloeobacter* xanthorhodopsin, but not in *Octadecabacter* xanthorhodopsin. This shows that the presence of an equivalent to Gly156 is not sufficient to enable binding of salinixanthin in poorly conserved keto-carotenoid binding sites and indicates that subgroup II members cannot bind keto-carotenoids.

The subgroup II xanthorhodopsins were predicted to be light-driven proton pumps like their subgroup I relatives, as they possess conserved residues that are indicative for this function (Supplementary Figure S8) [97]. To confirm this assumption the efflux of protons in xanthorhodopsin-producing *E. coli* cells was assessed via the acidification rate in a non-buffered, nutrient-free cell suspension according to Béjà et al. [19]. Suspensions of recombinant cells expressing *Octadecabacter* xanthorhodopsin showed a light-induced acidification, which was absent in suspensions without rhodopsins (Supplementary Figure S7). This result indicates that *Octadecabacter* xanthorhodopsins function as light-driven proton pumps. However, no growth advantage was observed for *Octadecabacter* cells in light compared to dark (data not shown). In several species, the function of proteorhodopsins is only supporting survival during periods of starvation [98]–[100]. Thus the activity of the *Octadecabacter* xanthorhodopsins might be required during transition periods with rapidly changing nutrient availabilities such as formation and melting of sea ice, or to overcome low diffusion rates of nutrients in sea ice.

### Conclusions

The genome analyses of the *Octadecabacter* strains emphasize the importance of horizontal gene transfer among members of the *Roseobacter* clade. This clade exhibits a large and accessible pan-genome, which seems to be more characteristic than its core-genome. GTAs enable individual *Roseobacters* to access this pan-genome [68], resulting in a high ability to adapt to changing environmental conditions. In both analyzed *Octadecabacter* strains, the adaptability is enhanced through TE-mediated genome plasticity, which is much higher than that of other *Roseobacter* clade members. This is indicated by the large number and size of RGPs in the *Octadecabacter* genomes (Figure 1), and the numerous genomic rearrangements that are evident in genome alignments (Figure 3). A linkage of this trait to the sea ice habitat of the *Octadecabacter* strains is in accordance with Collins and Deming [65], [66], who suggested that sea ice environments are hotspots for HGT in marine ecosystems. Thus, polar *Octadecabacter* strains may be a driving force of the genomic diversity in marine *Roseobacters*. In order to address whether this is a specific trait of polar *Octadecabacter* strains or not it is demanding to perform a corresponding analysis of non-polar *Octadecabacter* isolates in future experiments.

Many of the characteristic features found in the *Octadecabacter* genomes may represent adaptations to polar habitats. For example, the cyanophycin ligase of *O. arcticus* could present an advantage to polar marine and sea ice organisms, as nitrogen can be a significant limiting factor to prokaryotic heterotrophic production in polar surface waters during summer [101]. Furthermore, storage compounds in general can enhance the survival of organisms in rapidly changing environments such as sea ice [102]. Mercury reduction can be found in various organisms from polar and non-polar habitats [76], [103]. However, it could be of special significance in polar habitats because the Arctic and Antarctic region both act as sinks for atmospheric mercury leading to seasonal accumulations of mercury in these ecosystems [104]–[106]. An importance of mercury resistance in polar habitats is also indicated by the fact that the corresponding gene clusters are conserved in the Arctic and the Antarctic *Octadecabacter* strain but absent in many other *Roseobacter* clade members. The xanthorhodopsins of the *Octadecabacter* strains were also linked to sea ice habitats. Comparative analysis of these rhodopsins revealed the presence of two distinct xanthorhodopsin subgroups. Functional differences of the two subgroups were indicated by the observed habitat preference, organization of the gene clusters, and the potential for keto-carotenoid-binding. Metagenome analyses showed that none of the xanthorhodopsin subgroups represent a characteristic marine trait. The xanthorhodopsin-encoding genes found in the *Octadecabacter*-genomes are more typical for ice-associated rather than marine organisms.

The 16S rRNA sequence relationships of polar and non-polar *Octadecabacter* strains indicate a direct connection between bacterial populations of both poles (Figure 2). Results of the genome analyses support this hypothesis. Most characteristic gene clusters are remarkably conserved in both *Octadecabacter* strains, despite the fact that they seem to originate from HGT. The most prominent examples are the xanthorhodopsin, flagella and gas vesicle gene clusters. Variations of these features found in mesophilic *Roseobacter* clade members are often highly divergent in sequence and/or organization (Supplementary Figures S4), indicating that the Arctic and the Antarctic *Octadecabacter* strains share a common distinct gene pool. Due to the psychrophilic lifestyle of the polar *Octadecabacter* strains, a transit over the warm surface waters of the equator is unlikely. However, cold deep-water currents could be a possible vector for transport of bacteria between both poles [9]. This assumption remains to be validated by community analyses along deep-water currents and polar surface waters.

## Supporting Information

Figure S1 **Neighbor-joining tree based on multi locus sequence analyses (MLSA).**
(PDF)

Figure S2 **Selected gene clusters that represent differences between the** ***Octadecabacter*** **strains.**
(PDF)

Figure S3 **Neighbor-Joining tree of cyanophycin ligases and cyanophycin ligase-like proteins.**
(PDF)

Figure S4 **Selected gene clusters shared by both** ***Octadecabacter*** **strains.**
(PDF)

Figure S5 **Conservation of subgroup I and subgroup II xanthorhodopsin gene clusters.**
(PDF)

Figure S6 **Abundance and diversity of rhodopsins in Sanger sequencing-based metagenomes.**
(PDF)

Figure S7 **Functional characterization of the O** ***ctadecabacter*** **xanthorhodopsins.**
(PDF)

Figure S8 **Functional residues important for proton translocation in different rhodopsins.**
(PDF)

Table S1 **Rhodopsin sequences used for phylogenetic and metagenomic analyses.**
(PDF)

Table S2 **Cyanophycin ligase and cyanophycin ligase-like protein sequences used for phylogenetic analyses.**
(PDF)

Table S3 **List of metagenome projects used for the analyses of biogeography and diversity of microbial rhodopsins.**
(PDF)

Table S4 **Overview of selected features in regions of enhanced genome plasticity in the genomes of** ***Octadecabacter arcticus*** **(Oar-RGP1-17, pOAR118, pOAR160) and** ***Octadecabacter antarcticus*** **(Oan-RGP1-16, pOAN63).**
(PDF)
