# Supplementary material for: Poles Apart: Arctic and Antarctic Octadecabacter strains Share High Genome Plasticity and a New Type of Xanthorhodopsin
Source: PLoS One. 2013 May 6;8(5):e63422. doi: 10.1371/journal.pone.0063422 (PMC3646047; doi:10.1371/journal.pone.0063422)
Supplement: Figure S1 — Neighbor-joining tree based on multi locus sequence analyses (MLSA). (PDF) [file pone.0063422.s001.pdf]

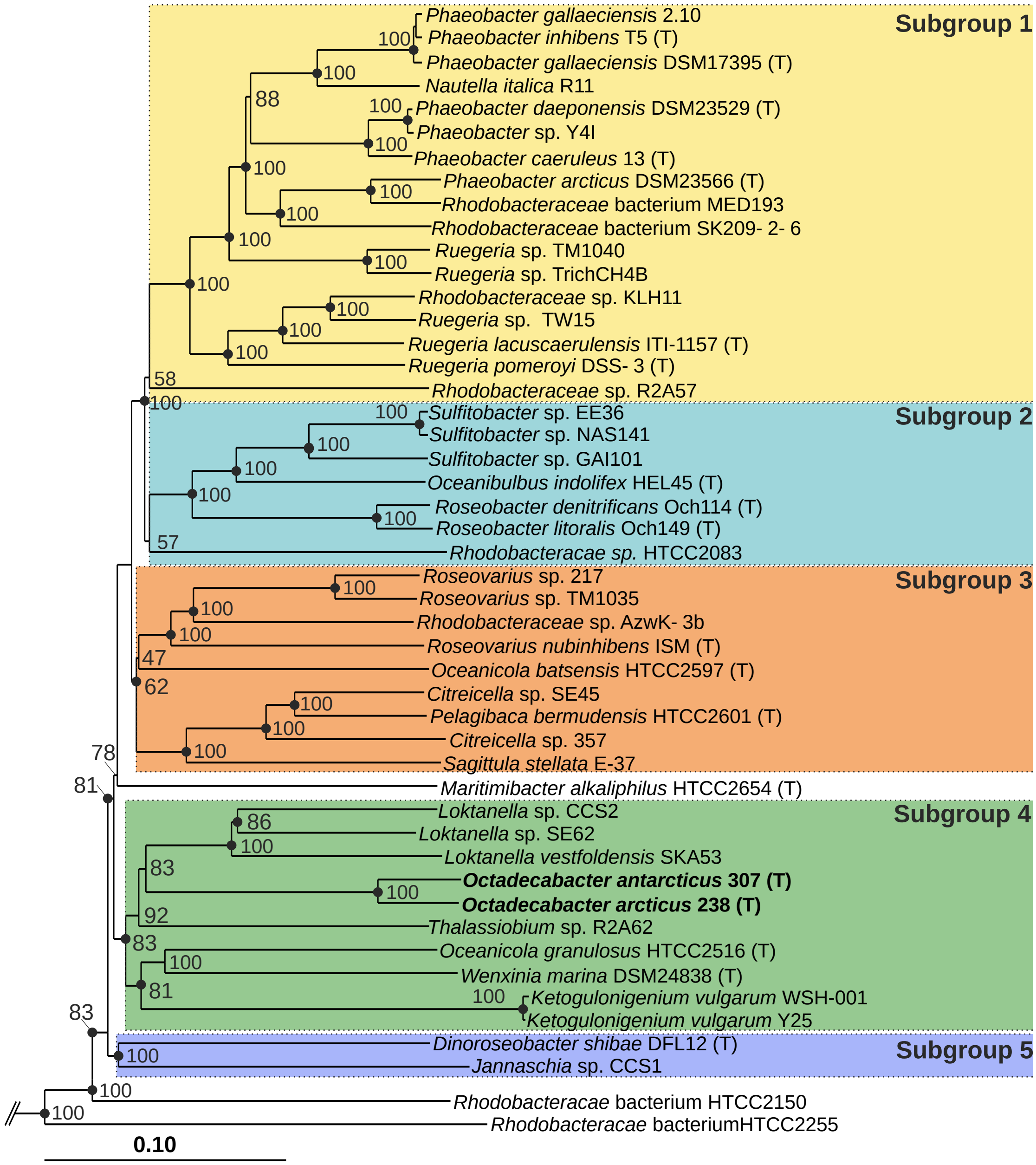

Figure S1. Neighbor-joining tree based on multi locus sequence analyses (MLSA). The tree was constructed using ARB v5.1 and the neighbor-joining algorithm. The amino acid sequences of single copy gene products of the *Roseobacter* and *Escherichia coli* core genome were concatenated, aligned and used for tree calculation. Bootstrap values are given for each node. Nodes that were reproduced by maximum-likelihood calculations are marked by black filled circles. Subclades of the *Roseobacter* clade, which were defined by Newton et al. (2010) [1], are marked by different colors. *E. coli* K12 MG1655 was used as outgroup. Type strains are marked with (T).

Reference:

1. Newton RJ, Griffin LE, Bowles KM, Meile C, Gifford S, et al. (2010) Genome characteristics of a generalist marine bacterial lineage. ISME J 4: 784-798.
