# Supplementary material for: Poles Apart: Arctic and Antarctic Octadecabacter strains Share High Genome Plasticity and a New Type of Xanthorhodopsin
Source: PLoS One. 2013 May 6;8(5):e63422. doi: 10.1371/journal.pone.0063422 (PMC3646047; doi:10.1371/journal.pone.0063422)
Supplement: Figure S2 — Selected gene clusters that represent differences between the Octadecabacter strains. (PDF) [file pone.0063422.s002.pdf]

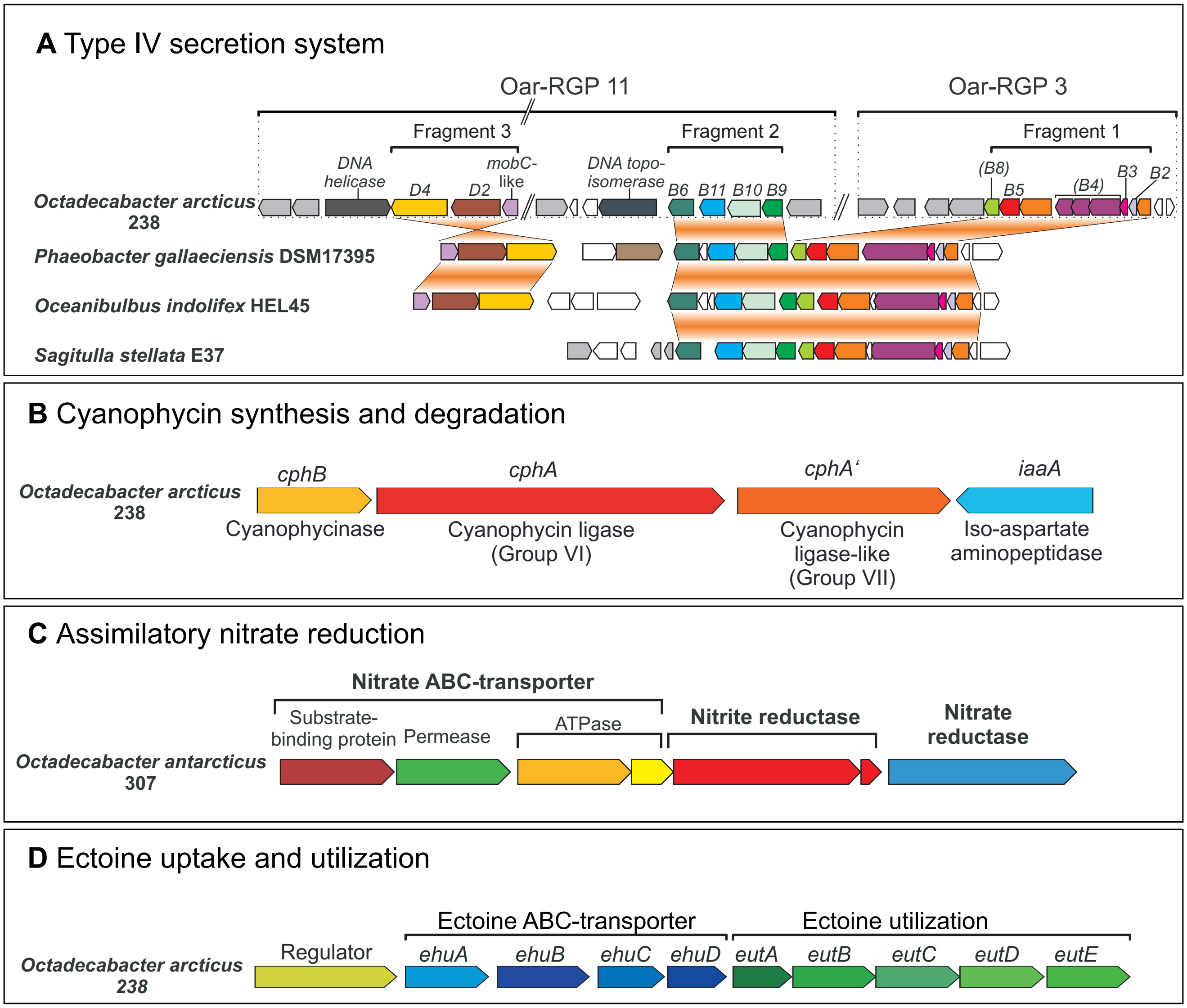

Figure S2. Selected gene clusters that represent differences between the *Octadecabacter* strains.

Genome sequences were obtained from the NCBI GenBank sequence database. (A) Transposable elements are indicated by grey and hypothetical proteins by white coloring. All other colors indicate orthologs in reference strains. The corresponding region of enhanced genome plasticity (RGP) that contains each gene cluster fragment is given above the gene symbols. The genes *virD2* and *virD4* are annotated as D2 and D4, the genes *virB2-virB11* as B2-B11, respectively. The genes *virB4* and *virB8* are fragmented in *O. arcticus* 238. The locus tags of the depicted genes are: OA238_c09340-OA238_c09420 (*Octadecabacter arcticus* 238, fragment 1), OA238_c29140-OA238_c29170 (*O. arcticus* 238, fragment 2), OA238_c29370-OA238_c29390 (*O. arcticus 238*, fragment 3), PGA1_c22800-PGA1_c22990 (*Phaeobacter gallaeciensis* DSM17395), OIHEL45_18196-OIHEL45_18306 (*Oceanibulbus indolifex* HEL45), SSE37_01595-SSE37_1505 (*Sagittula stellata* E-37). (B-C) The locus tags of the depicted genes are OA238_c34270-OA238_c34300 (B), OAN307_c02450-OAN307_c02510 (C), OA238_c23600-OA238_c23680 (D).
