# Supplementary material for: Poles Apart: Arctic and Antarctic Octadecabacter strains Share High Genome Plasticity and a New Type of Xanthorhodopsin
Source: PLoS One. 2013 May 6;8(5):e63422. doi: 10.1371/journal.pone.0063422 (PMC3646047; doi:10.1371/journal.pone.0063422)
Supplement: Figure S3 — Neighbor-Joining tree of cyanophycin ligases and cyanophycin ligase-like proteins. (PDF) [file pone.0063422.s003.pdf]

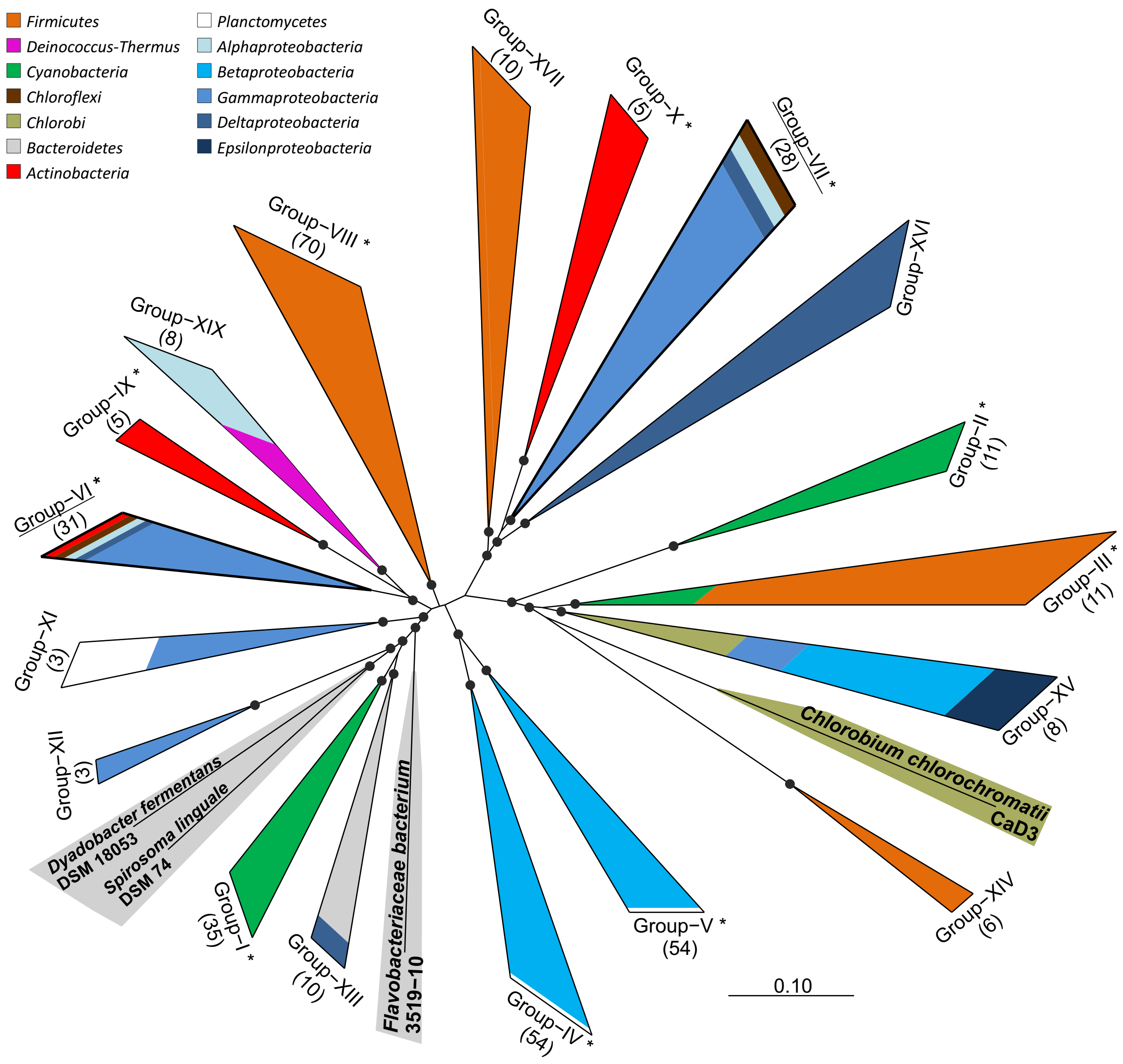


**Figure S3. Neighbor-Joining tree of cyanophycin ligases and cyanophycin ligase-like proteins.**

Nodes with Bootstrap values >50% are marked with black filled circles. Groups VI and VII, which contain the cyanophycin ligase and cyanophycin ligase-like protein of *Octadecabacter arcticus* 238 respectively, are underlined. The taxonomic composition of organisms represented by each group is indicated by different color proportions. The number of sequences in each group is given in parentheses. Groups that were originally described by Füser and Steinbüchel [1] are marked with an asterisk (*).

**Reference**


1. Füser G, Steinbüchel A. (2007) Analysis of genome sequences for genes of cyanophycin metabolism: Identifying putative cyanophycin metabolizing prokaryotes. Macromol Biosci 7: 278-296.
