# Supplementary material for: Poles Apart: Arctic and Antarctic Octadecabacter strains Share High Genome Plasticity and a New Type of Xanthorhodopsin
Source: PLoS One. 2013 May 6;8(5):e63422. doi: 10.1371/journal.pone.0063422 (PMC3646047; doi:10.1371/journal.pone.0063422)
Supplement: Figure S4 — Selected gene clusters shared by both Octadecabacter strains. (PDF) [file pone.0063422.s004.pdf]

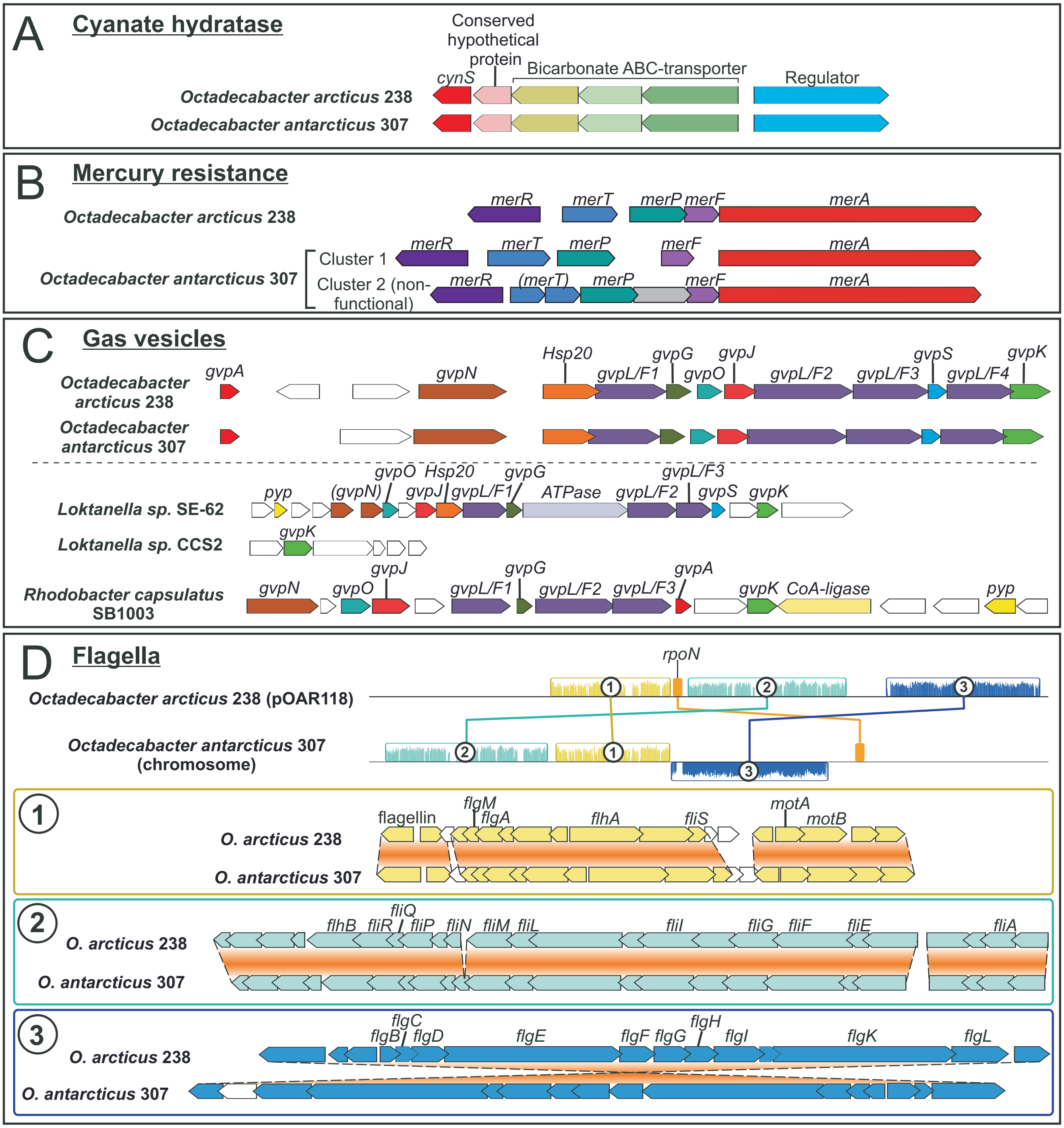

Figure S4. Selected gene clusters shared by both *Octadecabacter* strains.
Transposable elements are indicated by grey and hypothetical proteins by white coloring. All other colors indicate orthologs in reference strains. The genome sequence of *Loktanella* SE-62 was obtained from the J. Craig Venter Institute, all other sequences were obtained from the NCBI GenBank sequence database. The locus tags of the depicted genes are: OA238_c17620-c17670 (A, *Octadecabacter arcticus* 238), OAN307_c10320-c10370(A, *Octadecabacter antarcticus* 307), OA238_c45560-c45600 (B, *O. arcticus* 238), OAN307_c17630-c17670 and OAN307_c31790-c31850 (B, *O. antarcticus* 307), OA238_160p0640-160p0770 (C, *O. arcticus* 238), OAN307_c30330-c30450 (C, *O. antarcticus* 307), LSE62_2372-2390 (C, *Loktanella sp.* SE62), RCCS2_16386-16411 (C, *Loktanella sp.* CCS2), RCAP_rcc01051-rcc01067 (C, *Rhodobacter capsulatus* SB1003), OA238_118p0200-118p0780 (D, *O. arcticus* 238), OAN307_c29440-c30060 (D, *O. antarcticus* 307). In D, the relative orientation of the three flagella gene clusters in both organisms is shown as a Mauve [1] alignment on top. The organization of each gene cluster is shown beneath.

Reference

1. Darling A, Mau B, Perna N (2010). Progressive Mauve: Multiple Genome alignment with gene gain, loss and rearrangement. PLoS One 5: e11147.
