# Supplementary material for: Poles Apart: Arctic and Antarctic Octadecabacter strains Share High Genome Plasticity and a New Type of Xanthorhodopsin
Source: PLoS One. 2013 May 6;8(5):e63422. doi: 10.1371/journal.pone.0063422 (PMC3646047; doi:10.1371/journal.pone.0063422)
Supplement: Figure S5 — Conservation of subgroup I and subgroup II xanthorhodopsin gene clusters. (PDF) [file pone.0063422.s005.pdf]

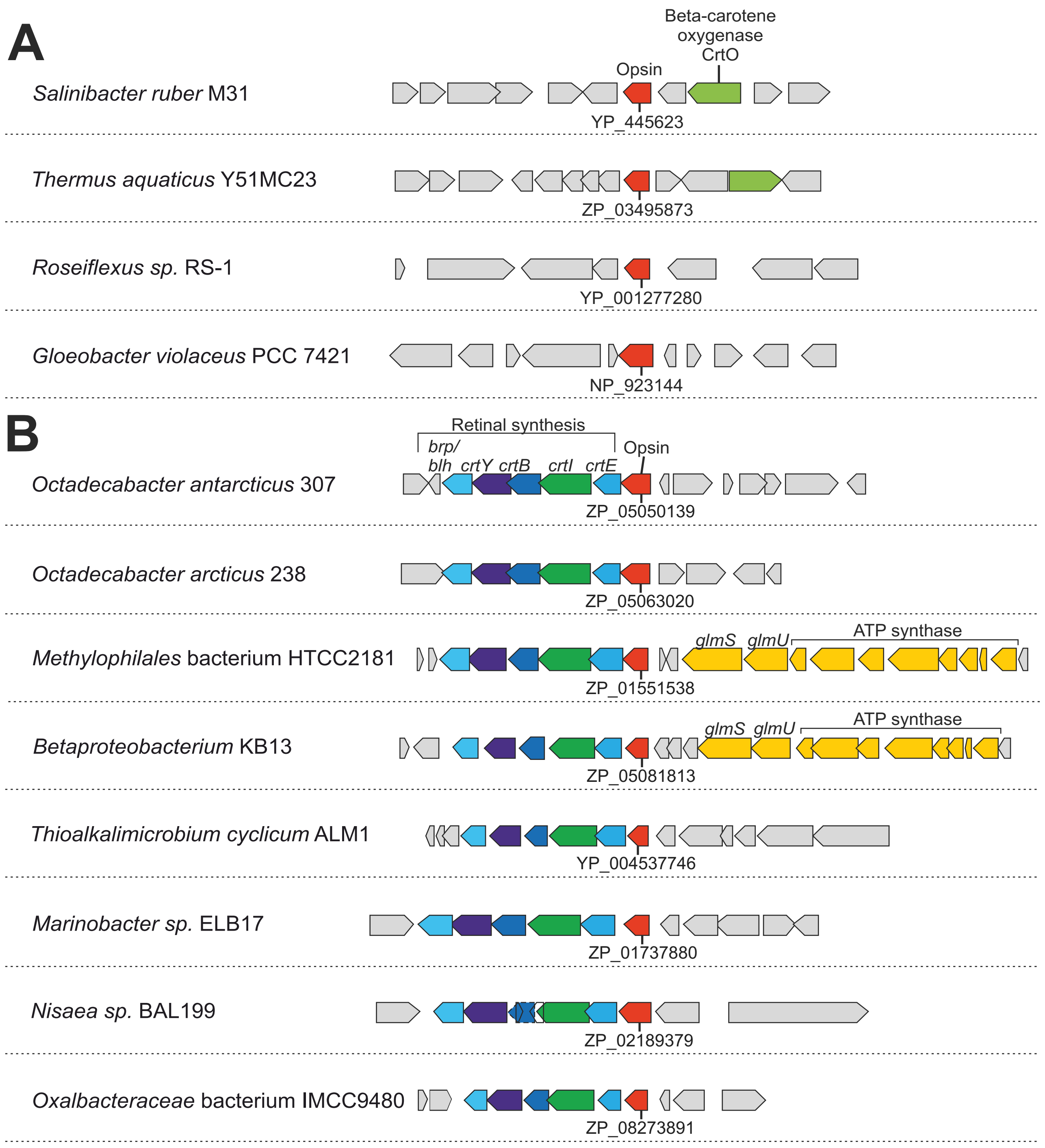

Figure S5. Conservation of subgroup I and subgroup II xanthorhodopsin gene clusters. Genome sequences were obtained from the NCBI GenBank sequence database. For each opsin gene the NCBI accession number of the corresponding gene product is given below the gene symbol. Genes depicted in grey show no homology between reference strains. All other colors indicate orthologs between reference strains. (A) Surrounding genes of subgroup I xanthorhodopsins . (B) Surrounding genes of subgroup II xanthorhodopsins. In *Nisaea* sp. BAL199, *crtB* is present, but has not been annotated. This is indicated by the dashed outline of the corresponding gene symbol. Transparent gene symbols indicate open reading frames, which have probably been wrongly annotated.
