# Supplementary material for: Poles Apart: Arctic and Antarctic Octadecabacter strains Share High Genome Plasticity and a New Type of Xanthorhodopsin
Source: PLoS One. 2013 May 6;8(5):e63422. doi: 10.1371/journal.pone.0063422 (PMC3646047; doi:10.1371/journal.pone.0063422)
Supplement: Figure S6 — Abundance and diversity of rhodopsins in Sanger sequencing-based metagenomes. (PDF) [file pone.0063422.s006.pdf]

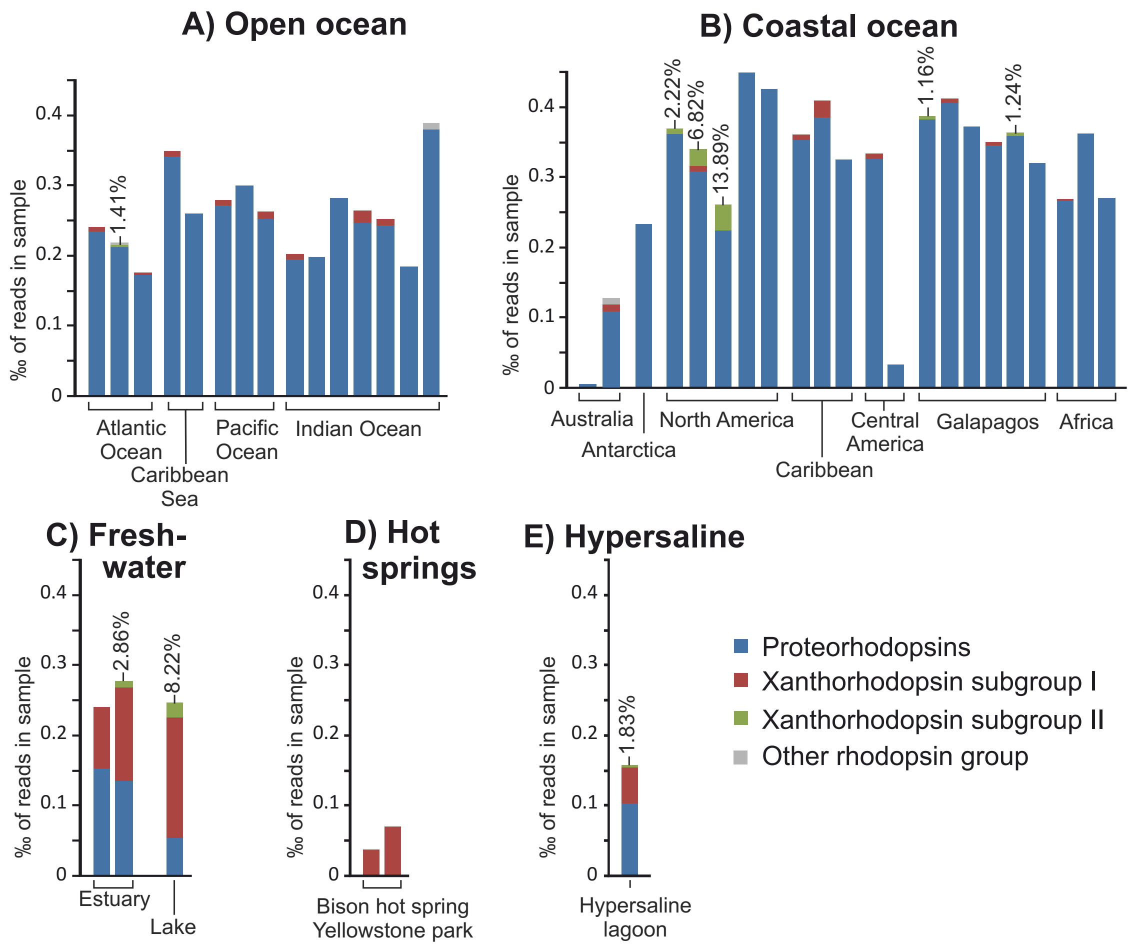

Figure S6. Abundance and diversity of rhodopsins in Sanger sequencing-based metagenomes.

The height of each bar indicates the normalized total abundance of rhodopsins, given in ‰ of reads in the respective metagenome sample. The relative abundances of different rhodopsin groups are indicated by the relative color proportions in each bar. The relative abundances of subgroup II xanthorhodopsins are given in percent above the respective bars. The represented metagenomes are listed in Supplementary Table S3.
