# Supplementary material for: Poles Apart: Arctic and Antarctic Octadecabacter strains Share High Genome Plasticity and a New Type of Xanthorhodopsin
Source: PLoS One. 2013 May 6;8(5):e63422. doi: 10.1371/journal.pone.0063422 (PMC3646047; doi:10.1371/journal.pone.0063422)
Supplement: Figure S7 — Functional characterization of the O ctadecabacter xanthorhodopsins. (PDF) [file pone.0063422.s007.pdf]

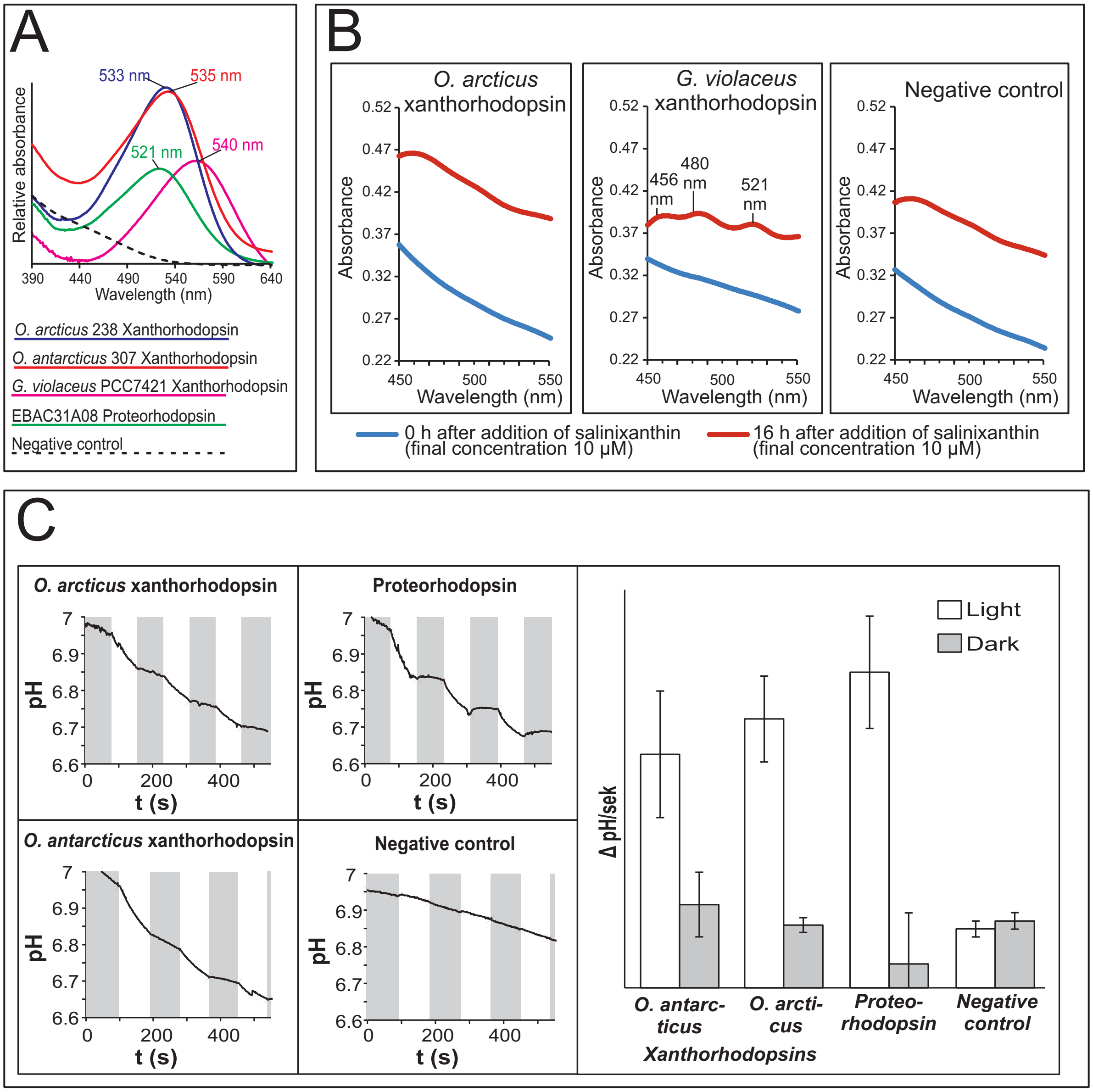

Figure S7. Functional characterization of the *Octadecabacter* xanthorhodopsins.
(A) Difference spectra of opsin-harboring membrane fragments after addition of 10 µM retinal. Spectra of different rhodopsin types are indicated by different colors. The absorption-maxima are given for each peak. (B) Difference spectra showing changes in the absorption spectra of rhodopsin harboring membrane fragment suspensions at different time points after addition of 10 µM salinixanthin. The difference spectra of *Gloeobacter* xanthorhodopsin harboring membrane fragments show changes at 456 nm, 480 nm and 521 nm. This indicates binding of salinixanthin by *Gloeobacter* xanthorhodopsin [1]. These absorption changes are not visible in membrane fragments bearing *O. arcticus* xanthorhodopsin or in the negative control. (C) Changes in pH value of recombinant *E. coli* cell suspensions expressing rhodopsins during alternating light/dark intervals. Left, line charts showing the change of the pH value over time. Light intervals are shown with white background, dark intervals are shaded in grey. A steady decline of the pH value could be observed in all cell suspensions, including the negative control. Cell suspensions expressing rhodopsins showed a temporarily increased acidification rate that was absent in the negative control. Right, averaged acidification rates of the different cell suspensions during light and dark intervals, respectively. The acidification rate is given as change of pH value per second. Indicators on each bar show the range of the respective standard error.

Reference

1. Imasheva ES, Balashov SP, Choi AR, Jung KH, Lanyi JK. (2009) Reconstitution of *Gloeobacter violaceus* rhodopsin with a light-harvesting carotenoid antenna. Biochemistry 48: 10948-10955.
