# Supplementary material for: Poles Apart: Arctic and Antarctic Octadecabacter strains Share High Genome Plasticity and a New Type of Xanthorhodopsin
Source: PLoS One. 2013 May 6;8(5):e63422. doi: 10.1371/journal.pone.0063422 (PMC3646047; doi:10.1371/journal.pone.0063422)
Supplement: Figure S8 — Functional residues important for proton translocation in different rhodopsins. (PDF) [file pone.0063422.s008.pdf]

A

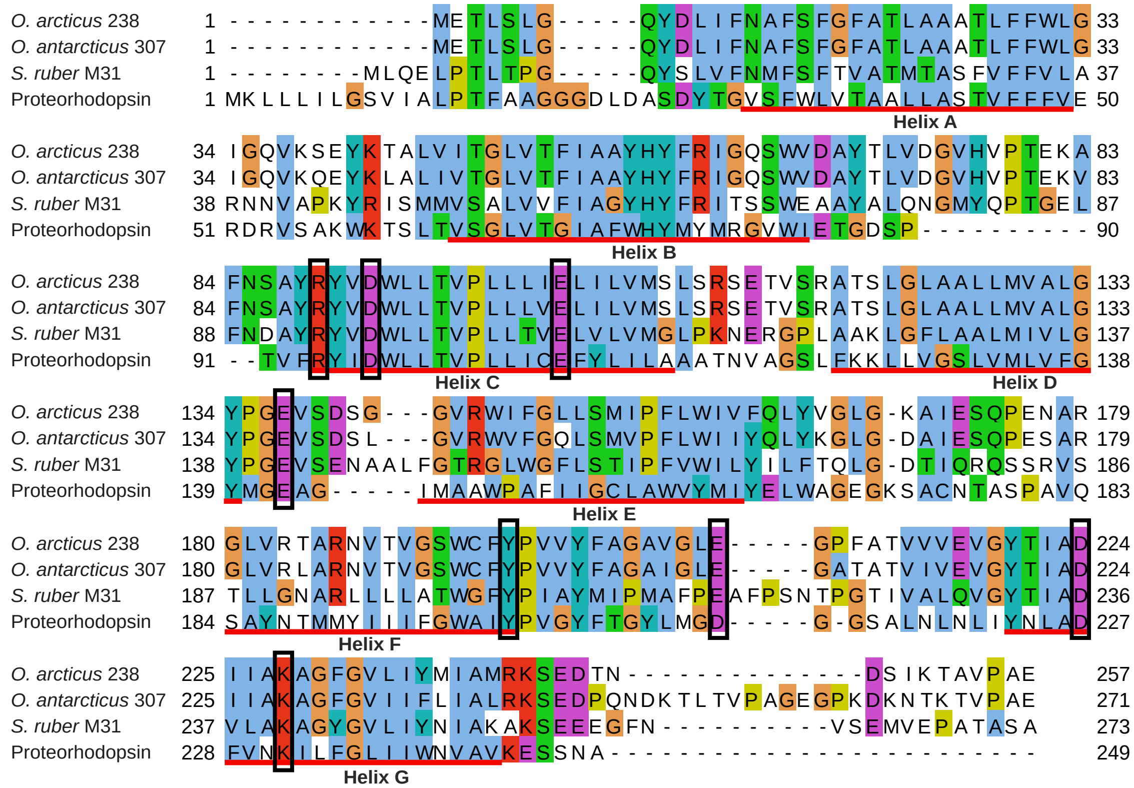

B

Rhodopsin source	Amino acid residue/sequence position							
O. arcticus 238 subgroup II xanthorhodopsin	Arg89	Asp92	Glu103	Glu137	Tyr196	Glu208	Asp224	Lys228
S. ruber M31 subgroup I xanthorhodopsin	Arg93	Asp96	Glu107	Glu141	Tyr203	Glu215	Asp236	Lys240
EBAC31A08 proteorhodopsin	Arg94	Asp97	Glu108	Glu142	Tyr200	Asp212	Asp227	Lys231
Function<br><br>[Reference]	Proton release complex<br><br>[1]	Proton acceptor<br><br>[1]	Proton donor<br><br>[2]	Proton release complex (suggested)<br><br>[3]	Proton pathway<br><br>[1]	Proton-Release-Complex<br><br>[1]	Counter-ion for Schiff-base<br><br>[1]	Schiff base<br><br>[1]
Corresponding residue in bacteriorhodopsin	Arg82	Asp85	Asp96	-	Tyr185	Glu194	Asp212	Lys216

Figure S8. Functional residues important for proton translocation in different rhodopsins.

(A) Alignment of subgroup I xanthorhodopsin from *S. ruber* M31, subgroup II xanthorhodopsins from the *Octadecabacter* strains and proteorhodopsin from the environmental clone EBAC31A08. The transmembrane helices of proteorhodopsin are marked as red lines beneath the alignment. Conserved residues with predicted functions for proton translocation are marked by black boxes. (B) Residues with predicted functions for proton translocation found in xanthorhodopsins and proteorhodopsins. The individual function is stated for each residue together with the corresponding reference. Sequence positions of equivalent residues in bacteriorhodopsin are given below.

References

1. Balashov SP (2000) Protonation reactions and their coupling in bacteriorhodopsin. Biochim Biophys Acta Bioenerg 1460: 75-94.
2. Balashov SP, Imasheva ES, Boichenko VA, Anton J, Wang JM, et al. (2005) Xanthorhodopsin: a proton pump with a light-harvesting carotenoid antenna. Science 309: 2061-2064.
3. Beja O, Aravind L, Koonin EV, Suzuki MT, Hadd A, et al. (2000) Bacterial rhodopsin: Evidence for a new type of phototrophy in the sea. Science 289: 1902-1906.
