# Supplementary material for: Poles Apart: Arctic and Antarctic Octadecabacter strains Share High Genome Plasticity and a New Type of Xanthorhodopsin
Source: PLoS One. 2013 May 6;8(5):e63422. doi: 10.1371/journal.pone.0063422 (PMC3646047; doi:10.1371/journal.pone.0063422)
Supplement: Table S1 — Rhodopsin sequences used for phylogenetic and metagenomic analyses. (PDF) [file pone.0063422.s009.pdf]

Table S1. Rhodopsin sequences used for phylogenetic and metagenomic analyses.

NCBI-Acc:	Organism	Strain/clone	Group	Blast-query
ZP_01049273	*Dokdonia donghaensis*	MED134	Proteorhodopsin	
ACM89772	*Dokdonia donghaensis*	PRO95	Proteorhodopsin	x
YP_002885111	*Exiguobacterium sp.*	AT1b	Proteorhodopsin	x
AAK30187	uncultured bacterium	MB 40m5	Proteorhodopsin	
ZP_05069347	*Candidatus Pelagibacter*	HTCC7211	Proteorhodopsin	
AAR05251	uncultured marine bacterium	fosmid ANT32C12	Proteorhodopsin	
AAG10475	uncultured marine bacterium	BAC EBAC31A08	Proteorhodopsin	
AAK30177	uncultured bacterium	HOT 75m1	Proteorhodopsin	
AAK30200	uncultured bacterium	PalE6	Proteorhodopsin	
EAQ40925	*Polaribacter sp.*	MED152	Proteorhodopsin	
ZP_01161099	*Photobacterium sp.*	SKA34	Proteorhodopsin	
ZP_02194911	*Vibrio sp.*	AND4	Proteorhodopsin	
ABR27802	*Candidatus Pelagibacter*	HTCC1062	Proteorhodopsin	
ABO88140	*Gammaproteobacterium*	HTCC2207	Proteorhodopsin	
ZP_01236264	*Photobacterium angustum*	S14	Proteorhodopsin	x
AAK30174	uncultured bacterium	BAC EBAC40E8	Proteorhodopsin	
AAK30175	uncultured bacterium	BAC EBAC64A5	Proteorhodopsin	
AAK30176	uncultured bacterium	HOT 0m1	Proteorhodopsin	
AAK30178	uncultured bacterium	HOT 75m3	Proteorhodopsin	
AAK30180	uncultured bacterium	HOT 75m8	Proteorhodopsin	
AAK30181	uncultured bacterium	MB 0m1	Proteorhodopsin	
AAK30182	uncultured bacterium	MB 0m2	Proteorhodopsin	
AAK30184	uncultured bacterium	MB 20m5	Proteorhodopsin	
AAK30185	uncultured bacterium	MB 20m12	Proteorhodopsin	
AAK30186	uncultured bacterium	MB 40m1	Proteorhodopsin	
AAK30188	uncultured bacterium	MB 40m12	Proteorhodopsin	
AAK30189	uncultured bacterium	MB 100m5	Proteorhodopsin	x
AAK30190	uncultured bacterium	MB 100m7	Proteorhodopsin	
AAK30191	uncultured bacterium	MB 100m9	Proteorhodopsin	
AAK30192	uncultured bacterium	MB 100m10	Proteorhodopsin	
AAK30193	uncultured bacterium	PalB1	Proteorhodopsin	
AAK30194	uncultured bacterium	PalB2	Proteorhodopsin	
AAK30195	uncultured bacterium	PalB5	Proteorhodopsin	
AAK30196	uncultured bacterium	PalB6	Proteorhodopsin	
AAK30197	uncultured bacterium	PalB7	Proteorhodopsin	
AAK30198	uncultured bacterium	PalB8	Proteorhodopsin	
AAK30199	uncultured bacterium	PalE1	Proteorhodopsin	
AAK30201	uncultured bacterium	PalE7	Proteorhodopsin	
AAO21434	uncultured bacterium	MED26	Proteorhodopsin	
AAO21435	uncultured bacterium	MED27	Proteorhodopsin	
AAO21436	uncultured bacterium	MED36	Proteorhodopsin	
AAO21437	uncultured bacterium	MED101	Proteorhodopsin	
AAO21438	uncultured bacterium	MED102	Proteorhodopsin	

AAO21440	uncultured bacterium	MED25	Proteorhodopsin
AAO21441	uncultured bacterium	MED202	Proteorhodopsin
AAO21444	uncultured bacterium	REDA9	Proteorhodopsin
AAO21445	uncultured bacterium	REDB9	Proteorhodopsin
AAO21446	uncultured bacterium	REDF9	Proteorhodopsin
AAO21449	uncultured bacterium	RED23	Proteorhodopsin
AAO21451	uncultured bacterium	RED30	Proteorhodopsin
AAO21452	uncultured bacterium	RED4	Proteorhodopsin
AAO21453	uncultured bacterium	RED5	Proteorhodopsin
AAO21454	uncultured bacterium	REDr6a5a14	Proteorhodopsin
AAO21455	uncultured bacterium	REDr6a5a6	Proteorhodopsin
AAO73909	uncultured bacterium	REDs3_7	Proteorhodopsin
AAO73910	uncultured bacterium	REDr7_1_15	Proteorhodopsin
AAO73911	uncultured bacterium	REDs3_15	Proteorhodopsin
AAO73912	uncultured bacterium	medA15r8ex6	Proteorhodopsin
AAO73913	uncultured bacterium	REDr7_1_16	Proteorhodopsin
AAO73914	uncultured bacterium	medA15r11b9	Proteorhodopsin
AAO73915	uncultured bacterium	medA15r9b5	Proteorhodopsin
AAO73916	uncultured bacterium	medA15r8b3	Proteorhodopsin
AAO73917	uncultured bacterium	medA15r11b3	Proteorhodopsin
AAO73918	uncultured bacterium	medA15_r8_1	Proteorhodopsin
AAO73919	uncultured bacterium	medA17R9_1	Proteorhodopsin
AAO73920	uncultured bacterium	medA15r8b9	Proteorhodopsin
AAO73921	uncultured bacterium	medA19_R8_16	Proteorhodopsin
AAO73922	uncultured bacterium	medA19_R8_19	Proteorhodopsin
AAO73923	uncultured bacterium	medA17_R8_6	Proteorhodopsin
AAO73924	uncultured bacterium	medA15r9b7	Proteorhodopsin
AAO73925	uncultured bacterium	medA15_R8_3	Proteorhodopsin
AAO73926	uncultured bacterium	medA15r10b5	Proteorhodopsin
AAO73927	uncultured bacterium	medA19_r9_9	Proteorhodopsin
AAO73928	uncultured bacterium	medA15_r8ex7	Proteorhodopsin
AAO73929	uncultured bacterium	medA19_R8_20	Proteorhodopsin
AAO73931	uncultured bacterium	medA15_r9_3	Proteorhodopsin
AAT09899	uncultured bacterium	N2_R17_6	Proteorhodopsin
AAT09900	uncultured bacterium	ANT131_R1_4	Proteorhodopsin
AAT09902	uncultured bacterium	A15_R9EX2	Proteorhodopsin
AAT09903	uncultured bacterium	MEDA15r9b9	Proteorhodopsin
AAT12291	uncultured bacterium	REDr7a3a5	Proteorhodopsin
AAT38634	uncultured bacterium	NA13_R15_2	Proteorhodopsin
AAY68055	uncultured bacterium	MEDPR46A6	Proteorhodopsin
AAY68065	uncultured bacterium	MEDPR66A3	Proteorhodopsin
AAY82613	uncultured bacterium	MedeBAC35C06	Proteorhodopsin
ABL97257	uncultured marine bacterium	EB0_50A10	Proteorhodopsin
ABL97415	uncultured marine bacterium	EB80_02D08	Proteorhodopsin
ABL97630	uncultured marine bacterium	EB0_39H12	Proteorhodopsin
ABL97764	uncultured marine bacterium	EB0_41B09	Proteorhodopsin

ABL97827	uncultured marine bacterium	HF10_49E08	Proteorhodopsin	
ADI16288	uncultured bacterium	HF0010_16H03	Proteorhodopsin	
Q9AFF7	*Gammaproteobacterium*	Hot 75m4	Proteorhodopsin	
YP_003552453	*Candidatus Puniceispirillum*	IMCC1322	Proteorhodopsin	
ZP_01447408	*Rhodobacterales bacterium*	HTCC2255	Proteorhodopsin	
ZP_01616930	*marine gamma*	HTCC2143	Proteorhodopsin	
ZP_01734914	*Flavobacteria bacterium*	BAL38	Proteorhodopsin	
ZP_07741624	*Vibrio caribbenthicus*	ATCC BAA-2122	Proteorhodopsin	
YP_001813902	*Exiguobacterium sibiricum*	255-15	Proteorhodopsin	
ABL60988	uncultured marine bacterium	HF10_19P19	Proteorhodopsin	
AAY82724	uncultured bacterium	MedeBAC86H08_1	Proteorhodopsin	x
ZP_01253360	*Psychroflexus torquis*	ATCC 700755	Proteorhodopsin	
YP_445623	*Salinibacter ruber*	M31	Subgroup I-XR	x
NP_923144	*Gloeobacter violaceus*	PCC 7421	Subgroup I-XR	
ACN42852	*Actinobacterium sp.*	MWH-EgelM2-3.D6	Subgroup I-XR	
YP_001277280	*Roseiflexus sp.*	RS-1	Subgroup I-XR	
ZP_03495873	*Thermus aquaticus*	Y51MC23	Subgroup I-XR	
ACN42850	*Candidatus Aquiluna*	MWH-Dar4	Subgroup I-XR	
ACN42851	*Candidatus Rhodoluna*	MWH-Ta8	Subgroup I-XR	
ACN42848	*Actinobacterium MWH-Uga1*	MWH-Uga1	Subgroup I-XR	
ACN42849	*Candidatus Rhodoluna*	MWH-Dar1	Subgroup I-XR	x
ZP_05050139	*Octadecabacter antarcticus*	307	Subgroup II-XR	
ZP_05063020	*Octadecabacter arcticus*	238	Subgroup II-XR	x
AAO14677	*Pyrocystis lunula*		Subgroup II-XR	
ZP_02189379	*Nisaea sp.*	BAL199	Subgroup II-XR	
ABV22426	*Oxyrrhis marina*	Om-5p-3-1	Subgroup II-XR	
ZP_01551538	*Methylophilales bacterium*	HTCC2181	Subgroup II-XR	
ZP_01737880	*Marinobacter sp.*	ELB17	Subgroup II-XR	
ABV22432	*Oxyrrhis marina*	Om-5p-3-7	Subgroup II-XR	
ABV22427	*Oxyrrhis marina*	Om-5p-3-2	Subgroup II-XR	
ABV22430	*Oxyrrhis marina*	Om-5p-3-5	Subgroup II-XR	
ZP_05081813	*Betaproteobacterium*	KB13	Subgroup II-XR	x
ABL97763	uncultured marine bacterium	EB0_41B09	Subgroup II-XR	
ZP_08273891	*Oxalobacteraceae bacterium*	IMCC9480	Subgroup II-XR	
YP_004537746	*Thioalkalimicrobium cyclicum*	ALM1	Subgroup II-XR	
AEF32712	*Polarella glacialis*	CCMP 2088	Subgroup II-XR	
AEF32711	*Polarella glacialis*		Subgroup II-XR	
YP_001689404	*Halobacterium salinarum*	R1	Bacteriorhodopsin	
YP_137573	*Haloarcula marismortui*	ATCC 43049	Bacteriorhodopsin	
ABT17417	*Halorubrum sp.*	TP009	Bacteriorhodopsin	
AAS15567	*Halorubrum xinjiangense*	BD-1	Bacteriorhodopsin	x
Q57101	*Haloarcula argentinensis*	arg-1	Bacteriorhodopsin	
BAA81816	*Haloarcula japonica*	TR-1	Bacteriorhodopsin	
P33971	*Halobacterium salinarum*	Port	Bacteriorhodopsin	
YP_003177111	*Halomicrobium mukohataei*	DSM 12286	Bacteriorhodopsin	x
YP_003129235	*Halorhabdus utahensis*	DSM 12940	Bacteriorhodopsin	

AAG42454	*Halobacterium salinarum*	XZ515	Bacteriorhodopsin	
ABT17372	uncultured *haloarchaeon*	FLAS10H9	Bacteriorhodopsin	
P29563	*Halobacterium sp.*	AUS-2	Bacteriorhodopsin	
P33972	*Halobacterium salinarum*	Shark	Bacteriorhodopsin	
P69051	*Halobacterium sp.*	aus-1	Bacteriorhodopsin	
P96787	*Halorubrum sodomense*		Bacteriorhodopsin	
YP_656801	*Haloquadratum walsbyi*	DSM 16790	Bacteriorhodopsin	x
AAU04564	*Halobiforma lacisalsi*	AJ5	Bacteriorhodopsin	
YP_136278	*Haloarcula marismortui*	ATCC 43049	Halorhodopsin	
BAA07823	*Halobacterium salinarum*	port	Halorhodopsin	
O93742	*Halorubrum sodomense*		Halorhodopsin	
YP_003176891	*Halomicrobium mukohataei*	DSM 12286	Halorhodopsin	
P33742	*Halobacterium sp.*	SG1	Halorhodopsin	
YP_002565246	*Halorubrum lacusprofundi*	ATCC 49239	Halorhodopsin	
BAA75201	*Haloterrigena sp.*	arg-4	Halorhodopsin	
NP_279315	*Halobacterium sp.*	NRC-1	Halorhodopsin	x
O93741	*Haloterrigena sp.*	arg-4	Halorhodopsin	
P15647	*Natronomonas pharaonis*		Halorhodopsin	
P94853	*Haloarcula vallismortis*	J. F. 54	Halorhodopsin	
Q48314	*Halobacterium salinarum*	Shark	Halorhodopsin	
YP_003480703	*Natrialba magadii*	ATCC 43099	Halorhodopsin	x
YP_325981	*Natronomonas pharaonis*	DSM 2160	Halorhodopsin	
YP_446872	*Salinibacter ruber*	M31	Halorhodopsin	
YP_658762	*Haloquadratum walsbyi*	DSM 16790	Halorhodopsin	
Q48334	*Haloarcula vallismortis*	J. F. 54	Sensory rhodopsin	
AAA72316	*Halobacterium salinarum*	Flx5R	Sensory rhodopsin	
AAC44370	*Halobacterium salinarum*	Flx15	Sensory rhodopsin	
YP_331142	*Natronomonas pharaonis*	DSM 2160	Sensory rhodopsin	x
BAB86796	*Halobacterium sp.*	aus-1	Sensory rhodopsin	x
YP_003572671	*Salinibacter ruber*	M8	Sensory rhodopsin	
YP_137680	*Haloarcula marismortui*	ATCC 43049	Sensory rhodopsin	x
YP_446677	*Salinibacter ruber*	M31	Sensory rhodopsin	
YP_446609	*Salinibacter ruber*	M31	Sensory rhodopsin	x
NP_280508	*Halobacterium sp.*	NRC-1	Sensory rhodopsin	
YP_002564761	*Halorubrum lacusprofundi*	ATCC 49239	Sensory rhodopsin	
XP_959421	*Neurospora crassa*	OR74A	fungal rhodopsin	
CBI54495	*Sordaria macrospora*	k-hell	fungal rhodopsin	
AAG01180	*Leptosphaeria maculans*	M1	fungal rhodopsin	
XP_001597420	*Sclerotinia sclerotiorum*	1980	fungal rhodopsin	
AAK49108	*Coccidioides posadasii*		fungal rhodopsin	
AAL27088	*Coccidioides posadasii*		fungal rhodopsin	
AAQ75384	*Oryza sativa*		fungal rhodopsin	
BAH28809	*Bipolaris oryzae*		fungal rhodopsin	
CAR82401	*Gibberella fujikuroi*	FKMC1995	fungal rhodopsin	
CBF82866	*Aspergillus nidulans*	FGSC A4	fungal rhodopsin	
CBX95095	*Leptosphaeria maculans*	v23.1.3	fungal rhodopsin	

EEH04666	*Ajellomyces capsulatus*	G186AR	fungal rhodopsin	
EEH18597	*Paracoccidioides brasiliensis*	Pb03	fungal rhodopsin	
EEH44959	*Paracoccidioides brasiliensis*	Pb18	fungal rhodopsin	
EER40098	*Ajellomyces capsulatus*	H143	fungal rhodopsin	
EFQ91496	*Pyrenophora teres*	0-1	fungal rhodopsin	
XP_001217277	*Aspergillus terreus*	NIH2624	fungal rhodopsin	
XP_001227935	*Chaetomium globosum*	CBS 148.51	fungal rhodopsin	
XP_001240940	*Coccidioides immitis*	RS	fungal rhodopsin	
XP_001262778	*Neosartorya fischeri*	NRRL 181	fungal rhodopsin	
XP_001272404	*Aspergillus clavatus*	NRRL 1	fungal rhodopsin	
XP_001395233	*Aspergillus niger*	CBS 513.88	fungal rhodopsin	x
XP_001536854	*Ajellomyces capsulatus*	NAm1	fungal rhodopsin	
XP_001558822	*Botryotinia fuckeliana*	B05.10	fungal rhodopsin	
XP_001791479	*Phaeosphaeria nodorum*	SN15	fungal rhodopsin	
XP_001904282	*Podospora anserina*	S mat+	fungal rhodopsin	x
XP_001937307	*Pyrenophora tritici-repentis*	Pt-1C-BFP	fungal rhodopsin	
XP_002143235	*Penicillium marneffei*	ATCC 18224	fungal rhodopsin	
XP_002479531	*Talaromyces stipitatus*	ATCC 10500	fungal rhodopsin	
XP_002479532	*Talaromyces stipitatus*	ATCC 10500	fungal rhodopsin	
XP_002479534	*Talaromyces stipitatus*	ATCC 10500	fungal rhodopsin	
XP_002542856	*Uncinocarpus reesii*	1704	fungal rhodopsin	
XP_002620840	*Ajellomyces dermatitidis*	SLH14081	fungal rhodopsin	
XP_002849585	*Arthroderma otae*	CBS 113480	fungal rhodopsin	
XP_003043878	*Nectria haematococca*	77-13-4	fungal rhodopsin	
XP_003065238	*Coccidioides posadasii*	C735 delta SOWgp	fungal rhodopsin	
XP_387730	*Gibberella zeae*	PH-1	fungal rhodopsin	
XP_660965	*Aspergillus nidulans*	FGSC A4	fungal rhodopsin	
XP_746789	*Aspergillus fumigatus*	Af293	fungal rhodopsin	
ZP_01440547	*Fulvimarina pelagi*	HTCC2506	unclassified	
ZP_06860850	*Citromicrobium bathyomarinum*	JL354	unclassified	
YP_003705905	*Truepera radiovictrix*	DSM 17093	unclassified	
YP_003706581	*Truepera radiovictrix*	DSM 17093	unclassified	
YP_004429763	*Krokinobacter sp.*	4H-3-7-5	unclassified	
YP_003410608	*Geodermatophilus obscurus*	DSM 43160	unclassified	
YP_001361545	*Kineococcus radiotolerans*	SRS30216	unclassified	
ADV90266	*Thermochromatium tepidum*	ATCC 43061	unclassified	

XR, xanthorhodopsin

Sequences used as query in the primary blast-analysis of metagenomes are marked by an "x" in the corresponding column.
