# Supplementary material for: Poles Apart: Arctic and Antarctic Octadecabacter strains Share High Genome Plasticity and a New Type of Xanthorhodopsin
Source: PLoS One. 2013 May 6;8(5):e63422. doi: 10.1371/journal.pone.0063422 (PMC3646047; doi:10.1371/journal.pone.0063422)
Supplement: Table S2 — Cyanophycin ligase and cyanophycin ligase-like protein sequences used for phylogenetic analyses. (PDF) [file pone.0063422.s010.pdf]

Table S2. Cyanophycin ligase and cyanophycin ligase-like protein sequences used for phylogenetic analyses.

Organism	Phylum	Protein Acc (NCBI)	Group
Synechocystis sp. PCC 6803	Cyanobacteria	NP_441210	Group-I *
Anabaena variabilis ATCC 29413	Cyanobacteria	YP_322331	Group-I *
Crocosphaera watsonii WH 8501	Cyanobacteria	ZP_00519191	Group-I *
Gloeobacter violaceus PCC 7421	Cyanobacteria	BAC92267	Group-I *
Nostoc punctiforme PCC 73102	Cyanobacteria	YP_001869064	Group-I *
Nostoc sp. PCC 7120	Cyanobacteria	P58572	Group-I *
Synechococcus sp. MA19	Cyanobacteria	AAL56990	Group-I *
Synechococcus sp. PCC 7002	Cyanobacteria	YP_001735638	Group-I *
Geminocystis herdmanii PCC 6308	Cyanobacteria	P56947	Group-I *
Thermosynechococcus elongatus BP-1	Cyanobacteria	BAC09722	Group-I *
Trichodesmium erythraeum IMS101	Cyanobacteria	ABG51217	Group-I *
Chromohalobacter salexigens DSM 3043	Proteobacteria (Gammaproteobacteria)	ABE58470	Group-XI
Anabaena variabilis ATCC 29413	Cyanobacteria	YP_320856	Group-II *
Nostoc punctiforme PCC 73102	Cyanobacteria	YP_001865424	Group-II *
Nostoc sp. PCC 7120	Cyanobacteria	NP_484617	Group-II *
Anabaena variabilis ATCC 29413	Cyanobacteria	YP_320778	Group-III*
Nostoc punctiforme PCC 73102	Cyanobacteria	YP_001865872	Group-III*
Nostoc sp. PCC 7120	Cyanobacteria	BAB73478	Group-III*
Bordetella avium 197N	Proteobacteria (Betaproteobacteria)	YP_785755	Group-IV *
Bordetella bronchiseptica RB50	Proteobacteria (Betaproteobacteria)	NP_890119	Group-IV *
Bordetella pertussis Tohama I	Proteobacteria (Betaproteobacteria)	NP_880454	Group-IV *
Dechloromonas aromatica RCB	Proteobacteria (Betaproteobacteria)	YP_286464	Group-IV *
Methylibium petroleiphilum PM1	Proteobacteria (Betaproteobacteria)	YP_001020219	Group-IV *
Nitrosomonas europaea ATCC 19718	Proteobacteria (Betaproteobacteria)	NP_840996	Group-IV *
Nitrosomonas eutropha C91	Proteobacteria (Betaproteobacteria)	ABI59649	Group-IV *
Nitrosospira multiformis ATCC 25196	Proteobacteria (Betaproteobacteria)	ABB75543	Group-IV *
Polaromonas sp. JS666	Proteobacteria (Betaproteobacteria)	YP_548263	Group-IV *
Polaromonas naphthalenivorans CJ2	Proteobacteria (Betaproteobacteria)	AANM01000000 AANM010	Group-IV *
Ralstonia eutropha H16	Proteobacteria (Betaproteobacteria)	YP_725290	Group-IV *
Ralstonia eutropha JMP134	Proteobacteria (Betaproteobacteria)	AAZ61946	Group-IV *
Cupriavidus metallidurans CH34	Proteobacteria (Betaproteobacteria)	YP_582859	Group-IV *
Rhodoferax ferrireducens T118	Proteobacteria (Betaproteobacteria)	ABD69049	Group-IV *
Bordetella avium 197N	Proteobacteria (Betaproteobacteria)	YP_785754	Group-V *
Bordetella bronchiseptica RB50	Proteobacteria (Betaproteobacteria)	NP_890120	Group-V *
Bordetella parapertussis 12822	Proteobacteria (Betaproteobacteria)	NP_885354	Group-V *
Bordetella pertussis Tohama I	Proteobacteria (Betaproteobacteria)	NP_880453	Group-V *
Dechloromonas aromatica RCB	Proteobacteria (Betaproteobacteria)	YP_286465	Group-V *
Methylibium petroleiphilum PM1	Proteobacteria (Betaproteobacteria)	YP_001020220	Group-V *
Nitrosomonas europaea ATCC 19718	Proteobacteria (Betaproteobacteria)	NP_840997	Group-V *
Nitrosomonas eutropha C91	Proteobacteria (Betaproteobacteria)	ABI59650	Group-V *
Nitrosospira multiformis ATCC 25196	Proteobacteria (Betaproteobacteria)	ABB75542	Group-V *
Polaromonas sp. JS666	Proteobacteria (Betaproteobacteria)	YP_548262	Group-V *
Polaromonas naphthalenivorans CJ2	Proteobacteria (Betaproteobacteria)	AANM01000000 AANM010	Group-V *
Ralstonia eutropha H16	Proteobacteria (Betaproteobacteria)	YP_725289	Group-V *
Ralstonia eutropha JMP134	Proteobacteria (Betaproteobacteria)	AAZ61947	Group-V *
Cupriavidus metallidurans CH34	Proteobacteria (Betaproteobacteria)	YP_582858	Group-V *
Rhodoferax ferrireducens T118	Proteobacteria (Betaproteobacteria)	ABD69048	Group-V *
Acinetobacter sp. ADP1	Proteobacteria (Gammaproteobacteria)	CAG68152	Group-VI *
Mycobacterium marinum M	Actinobacteria	YP_001850711	Group-VI *
Colwellia psychrerythraea 34H	Proteobacteria (Gammaproteobacteria)	YP_268601	Group-VI *
Francisella tularensis subsp. tularensis SCHU S4	Proteobacteria (Gammaproteobacteria)	YP_170103	Group-VI *
Colwellia psychrerythraea 34H	Proteobacteria (Gammaproteobacteria)	YP_268602	Group-VII *
Francisella tularensis subsp. tularensis SCHU S4	Proteobacteria (Gammaproteobacteria)	YP_170102	Group-VII *
Clostridium beijerinckii NCIMB 8052	Firmicutes	ABR32581	Group-VIII *
Clostridium botulinum A str. ATCC 3502	Firmicutes	CAL81675	Group-VIII *
Clostridium perfringens ATCC 13124	Firmicutes	YP_696891	Group-VIII *
Clostridium perfringens str. 13	Firmicutes	NP_563129	Group-VIII *
Clostridium perfringens SM101	Firmicutes	YP_699487	Group-VIII *
Clostridium tetani E88	Firmicutes	NP_780989	Group-VIII *
Clostridium thermocellum ATCC 27405	Firmicutes	YP_001038599	Group-VIII *
Desulfitobacterium hafniense DCB-2	Firmicutes	YP_002456590	Group-VIII *
Syntrophomonas wolfei subsp. wolfei str. Goettinge	Firmicutes	ABI68658	Group-VIII *
Thermoanaerobacter pseudethanolicus ATCC 33223	Firmicutes	YP_001666236	Group-VIII *
Thermoanaerobacter tengcongensis MB4	Firmicutes	AAM25889	Group-VIII *
Janibacter sp. HTCC2649	Actinobacteria	ZP_00995355	Group-IX *
Janibacter sp. HTCC2649	Actinobacteria	ZP_00995358	Group-X *
Bordetella parapertussis 12822	Proteobacteria (Betaproteobacteria)	BPP3183	Group-IV *
Octadecabacter antarcticus 238	Proteobacteria (Alphaproteobacteria)	ZP_05067667	Group-VI *
Octadecabacter antarcticus 238	Proteobacteria (Alphaproteobacteria)	ZP_05064377	Group-VII *
Francisella novicida FTE	Proteobacteria (Gammaproteobacteria)	ZP_03057450	Group-VI *
Francisella novicida U112	Proteobacteria (Gammaproteobacteria)	YP_898752	Group-VI *
Francisella novicida GA99-3548	Proteobacteria (Gammaproteobacteria)	ZP_04990001	Group-VI *
Francisella tularensis subsp. tularensis WY96-3418	Proteobacteria (Gammaproteobacteria)	YP_001122090	Group-VI *
Francisella novicida FTG	Proteobacteria (Gammaproteobacteria)	ZP_03247111	Group-VI *
Francisella tularensis subsp. tularensis MA00-2987	Proteobacteria (Gammaproteobacteria)	ZP_03665784	Group-VI *
Francisella tularensis subsp. tularensis FSC198	Proteobacteria (Gammaproteobacteria)	YP_667235	Group-VI *
Francisella tularensis subsp. tularensis FSC033	Proteobacteria (Gammaproteobacteria)	ZP_04986699	Group-VI *
Francisella tularensis subsp. mediasiatica FSC147	Proteobacteria (Gammaproteobacteria)	YP_001891929	Group-VI *
Francisella tularensis subsp. tularensis MA00-2987	Proteobacteria (Gammaproteobacteria)	ZP_05247730	Group-VI *
Francisella novicida GA99-3549	Proteobacteria (Gammaproteobacteria)	ZP_04988557	Group-VI *
Francisella tularensis subsp. holarctica URFT1	Proteobacteria (Gammaproteobacteria)	ZP_06558955	Group-VI *
Francisella tularensis subsp. holarctica FTNF002-0	Proteobacteria (Gammaproteobacteria)	YP_001428312	Group-VI *

Francisella tularensis subsp. holarctica OSU18	Proteobacteria (Gammaproteobacteria)	YP_763387	Group-VI *
Francisella tularensis subsp. holarctica 257	Proteobacteria (Gammaproteobacteria)	ZP_04983545	Group-VI *
Francisella tularensis subsp. holarctica LVS	Proteobacteria (Gammaproteobacteria)	YP_513553	Group-VI *
Francisella tularensis subsp. holarctica FSC200	Proteobacteria (Gammaproteobacteria)	ZP_02274705	Group-VI *
Francisella tularensis subsp. holarctica FSC022	Proteobacteria (Gammaproteobacteria)	ZP_04985201	Group-VI *
Francisella philomiragia subsp. philomiragia ATCC	Proteobacteria (Gammaproteobacteria)	ZP_04755870	Group-VI *
Francisella philomiragia subsp. philomiragia ATCC	Proteobacteria (Gammaproteobacteria)	YP_001678220	Group-VI *
Francisella philomiragia subsp. philomiragia ATCC	Proteobacteria (Gammaproteobacteria)	ZP_05249534	Group-VI *
Congregibacter litoralis KT71	Proteobacteria (Gammaproteobacteria)	ZP_01104810	Group-VI *
gamma proteobacterium NOR5-3	Proteobacteria (Gammaproteobacteria)	ZP_05125957	Group-VI *
Plesiocystis pacifica SIR-1	Proteobacteria (Deltaproteobacteria)	ZP_01912973	Group-VI *
Acinetobacter sp. ADP1	Proteobacteria (Gammaproteobacteria)	YP_045974	Group-VI *
Herpetosiphon aurantiacus ATCC 23779	Chloroflexi	YP_001543393	Group-VI *
Deinococcus maricopensis DSM 21211	Deinococcus-Thermus	YP_004172426	Group-XIX
Deinococcus deserti VCD115	Deinococcus-Thermus	YP_002787896	Group-XIX
Nakamurella multipartita DSM 44233	Actinobacteria	YP_003204118	Group-IX *
Kribbella flavida DSM 17836	Actinobacteria	YP_003381562	Group-IX *
Deinococcus maricopensis DSM 21211	Deinococcus-Thermus	YP_004171029	Group-XIX
Dermacoccus sp. Ellin185	Actinobacteria	ZP_07705583	Group-IX *
Sphingobium chlorophenolicum L-1	Proteobacteria (Alphaproteobacteria)	ZP_07576634	Group-XIX
Rhizobium leguminosarum bv. trifolii WSM1325	Proteobacteria (Alphaproteobacteria)	YP_002979139	Group-XIX
Deinococcus deserti VCD115	Deinococcus-Thermus	YP_002787193	Group-XIX
Kytococcus sedentarius DSM 20547	Actinobacteria	YP_003149147	Group-IX *
Mesorhizobium ciceri biovar biserrulae WSM1271	Proteobacteria (Alphaproteobacteria)	YP_004134272	Group-XIX
Microcystis aeruginosa NIES-843	Cyanobacteria	YP_001657760	Group-I *
Gloeobacter violaceus PCC 7421	Cyanobacteria	NP_927272	Group-I *
Thermosinus carboxydivorans Nor1	Firmicutes	ZP_01666685	Group-VIII *
Synechococcus sp. JA-2-3B'a(2-13)	Cyanobacteria	YP_477153	Group-I *
Cyanothece sp. PCC 7822	Cyanobacteria	YP_003888052	Group-I *
Thermosediminibacter oceani DSM 16646	Firmicutes	YP_003824462	Group-VIII *
Flavobacteriales bacterium ALC-1	Bacteroidetes	ZP_02181016	Group-XIII
Flavobacteria bacterium BAL38	Bacteroidetes	ZP_01734516	Group-XIII
Cyanothece sp. ATCC 51142	Cyanobacteria	YP_001803653	Group-I *
Cyanothece sp. CCY0110	Cyanobacteria	ZP_01728704	Group-I *
Pedobacter sp. BAL39	Bacteroidetes	ZP_01883431	Group-XIII
Cyanothece sp. PCC 8801	Cyanobacteria	YP_002371193	Group-I *
Cyanothece sp. PCC 8802	Cyanobacteria	YP_003136756	Group-I *
Cyanothece sp. PCC 7424	Cyanobacteria	YP_002376197	Group-I *
Chryseobacterium gleum ATCC 35910	Bacteroidetes	ZP_07087614	Group-XIII
Flavobacterium psychrophilum JIP02/86	Bacteroidetes	YP_001296010	Group-XIII
Flavobacteriaceae bacterium 3519-10	Bacteroidetes	YP_003095875	Group-XIII
Nostoc sp. PCC 7120	Cyanobacteria	NP_487919	Group-I *
Kordia algicida OT-1	Bacteroidetes	ZP_02160983	Group-XIII
Thermoanaerobacter tengcongensis MB4	Firmicutes	NP_624285	Group-VIII *
Cyanothece sp. PCC 7425	Cyanobacteria	YP_002485516	Group-I *
Thermosynechococcus elongatus BP-1	Cyanobacteria	NP_682960	Group-I *
Mucilaginibacter paludis DSM 18603	Bacteroidetes	ZP_07750076	Group-XIII
Arthrospira maxima CS-328	Cyanobacteria	ZP_03273369	Group-I *
'Nostoc azollae' 0708	Cyanobacteria	YP_003720623	Group-I *
Trichodesmium erythraeum IMS101	Cyanobacteria	YP_721690	Group-I *
Thermoanaerobacter italicus Ab9	Firmicutes	YP_003478146	Group-VIII *
Thermoanaerobacterium xylanolyticum LX-11	Firmicutes	ZP_08117403	Group-VIII *
Thermoanaerobacter mathranii subsp. mathranii str.	Firmicutes	YP_003677944	Group-VIII *
Thermoanaerobacter sp. X513	Firmicutes	YP_003905284	Group-VIII *
Thermoanaerobacter sp. X514	Firmicutes	YP_001663993	Group-VIII *
Thermoanaerobacter sp. X561	Firmicutes	ZP_07131098	Group-VIII *
Arthrospira platensis str. Paraca	Cyanobacteria	ZP_06381780	Group-I *
Thermoanaerobacter brockii subsp. finnii Ako-1	Firmicutes	YP_004187209	Group-VIII *
Thermoanaerobacter ethanolicus JW 200	Firmicutes	ZP_08211938	Group-VIII *
Thermincola potens JR	Firmicutes	YP_003638920	Group-VIII *
Microcoleus chthonoplastes PCC 7420	Cyanobacteria	ZP_05030078	Group-I *
Desulfonatronospira thiodismutans ASO3-1	Proteobacteria (Deltaproteobacteria)	ZP_07015214	Group-XIII
Cylindrospermopsis raciborskii CS-505	Cyanobacteria	ZP_06307508	Group-I *
Lyngbya sp. PCC 8106	Cyanobacteria	ZP_01618966	Group-I *
Synechococcus sp. PCC 7335	Cyanobacteria	ZP_05035312	Group-I *
Thermoanaerobacterium thermosaccharolyticum DSM 57	Firmicutes	YP_003853266	Group-VIII *
Oscillatoria sp. PCC 6506	Cyanobacteria	ZP_07109007	Group-I *
Raphidiopsis brookii D9	Cyanobacteria	ZP_06304438	Group-I *
Carboxydibrachium pacificum DSM 12653	Firmicutes	ZP_05092727	Group-VIII *
Gramella forsetii KT0803	Bacteroidetes	YP_860789	Group-XIII
Thermoanaerobacter ethanolicus CCSD1	Firmicutes	ZP_05492349	Group-VIII *
Planctomyces maris DSM 8797	Planctomycetes	ZP_01851722	Group-XI
Alkaliphilus metalliredigens QYMF	Firmicutes	YP_001319094	Group-VIII *
Acetivibrio cellulolyticus CD2	Firmicutes	ZP_07328672	Group-VIII *
Alkaliphilus oremlandii OhILAs	Firmicutes	YP_001513638	Group-VIII *
Nodularia spumigena CCY9414	Cyanobacteria	ZP_01631862	Group-I *
Dyadobacter fermentans DSM 18053	Bacteroidetes	YP_003084947	-
Thermoanaerobacter wiegelii Rt8.B1	Firmicutes	ZP_07547010	Group-VIII *
Spirosoma linguale DSM 74	Bacteroidetes	YP_003388938	-
Chromohalobacter salexigens DSM 3043	Proteobacteria (Gammaproteobacteria)	YP_573169	Group-XI
Dethiobacter alkaliphilus AHT 1	Firmicutes	ZP_03734994	Group-VIII *
Clostridium thermocellum JW20	Firmicutes	ZP_06249861	Group-VIII *
Legionella longbeachae NSW150	Proteobacteria (Gammaproteobacteria)	YP_003455995	Group-XII
Legionella longbeachae D-4968	Proteobacteria (Gammaproteobacteria)	ZP_06187977	Group-XII
Syntrophobotulus glycolicus DSM 8271	Firmicutes	YP_004264561	Group-VIII *

Desulfitobacterium hafniense Y51	Firmicutes	YP_516379	Group-VIII *
Syntrophomonas wolfei subsp. wolfei str. Goettinge	Firmicutes	YP_754029	Group-VIII *
Clostridium papyrosolvens DSM 2782	Firmicutes	ZP_08192769	Group-VIII *
Clostridium cellulolyticum H10	Firmicutes	YP_002504500	Group-VIII *
Janthinobacterium sp. Marseille	Proteobacteria (Betaproteobacteria)	YP_001355246	Group-V *
Clostridium thermocellum DSM 2360	Firmicutes	ZP_05430613	Group-VIII *
Natranaerobius thermophilus JW/NM-WN-LF	Firmicutes	YP_001916242	Group-VIII *
Planctomyces limnophilus DSM 3776	Planctomycetes	YP_003630693	Group-V *
Legionella drancourtii LLAP12	Proteobacteria (Gammaproteobacteria)	ZP_05108586	Group-XII
Clostridium carboxidivorans P7	Firmicutes	ZP_05392782	Group-VIII *
Alicyclobacillus acidocaldarius subsp. acidocaldar	Firmicutes	YP_003183625	Group-VIII *
Bacillus sp. SG-1	Firmicutes	ZP_01861428	Group-XVIII
Flavobacteriaceae bacterium 3519-10	Bacteroidetes	YP_003096691	-
Rhodoferax ferrireducens T118	Proteobacteria (Betaproteobacteria)	YP_522579	Group-V *
Alicyclobacillus acidocaldarius LAA1	Firmicutes	ZP_03494771	Group-VIII *
Polynucleobacter necessarius subsp. asymbioticus Q	Proteobacteria (Betaproteobacteria)	YP_001155452	Group-V *
Clostridium botulinum B1 str. Okra	Firmicutes	YP_001779747	Group-VIII *
Nitrosomonas sp. AL212	Proteobacteria (Betaproteobacteria)	YP_004293563	Group-V *
Clostridium botulinum F str. Langeland	Firmicutes	YP_001389483	Group-VIII *
Clostridium botulinum A3 str. Loch Maree	Firmicutes	YP_001785449	Group-VIII *
Acidovorax delafieldii 2AN	Proteobacteria (Betaproteobacteria)	ZP_04761260	Group-V *
Ralstonia eutropha JMP134	Proteobacteria (Betaproteobacteria)	YP_296791	Group-V *
Laribacter hongkongensis HLHK9	Proteobacteria (Betaproteobacteria)	YP_002795963	Group-V *
Clostridium botulinum NCTC 2916	Firmicutes	ZP_02612116	Group-VIII *
Clostridium sporogenes ATCC 15579	Firmicutes	ZP_02993762	Group-VIII *
Clostridium botulinum A2 str. Kyoto	Firmicutes	YP_002802434	Group-VIII *
Clostridium botulinum Ba4 str. 657	Firmicutes	YP_002860990	Group-VIII *
Clostridium botulinum Bf	Firmicutes	ZP_02616935	Group-VIII *
Clostridium ljungdahlii DSM 13528	Firmicutes	YP_003778402	Group-VIII *
Clostridium botulinum A str. ATCC 3502	Firmicutes	YP_001252667	Group-VIII *
Clostridium botulinum A str. Hall	Firmicutes	YP_001386079	Group-VIII *
Clostridium botulinum A str. ATCC 19397	Firmicutes	YP_001382527	Group-VIII *
Candidatus Accumulibacter phosphatis clade IIA str	Proteobacteria (Betaproteobacteria)	YP_003169066	Group-V *
Leptothrix cholodnii SP-6	Proteobacteria (Betaproteobacteria)	YP_001793059	Group-V *
Gallionella capsiferriformans ES-2	Proteobacteria (Betaproteobacteria)	YP_003847386	Group-V *
Variovorax paradoxus EPS	Proteobacteria (Betaproteobacteria)	YP_004153456	Group-V *
Oxalobacteraceae bacterium IMCC9480	Proteobacteria (Betaproteobacteria)	ZP_08273977	Group-V *
Clostridium butyricum 5521	Firmicutes	ZP_02951534	Group-VIII *
Clostridium butyricum E4 str. BoNT E BL5262	Firmicutes	ZP_04526420	Group-VIII *
Nitrosospira multiformis ATCC 25196	Proteobacteria (Betaproteobacteria)	YP_412934	Group-V *
Herminiimonas arsenicoxydans	Proteobacteria (Betaproteobacteria)	YP_001101550	Group-V *
Variovorax paradoxus S110	Proteobacteria (Betaproteobacteria)	YP_002942975	Group-V *
Methylotenera sp. 301	Proteobacteria (Betaproteobacteria)	YP_003674411	Group-V *
Bacillus tusciae DSM 2912	Firmicutes	YP_003587985	Group-VIII *
Delftia acidovorans SPH-1	Proteobacteria (Betaproteobacteria)	YP_001566723	Group-V *
Nitrosomonas eutropha C91	Proteobacteria (Betaproteobacteria)	YP_747615	Group-V *
Cupriavidus taiwanensis LMG 19424	Proteobacteria (Betaproteobacteria)	YP_002004802	Group-V *
Herbaspirillum seropedicae SmR1	Proteobacteria (Betaproteobacteria)	YP_003775577	Group-V *
Achromobacter piechaudii ATCC 43553	Proteobacteria (Betaproteobacteria)	ZP_06686558	Group-V *
Verminephrobacter eiseniae EF01-2	Proteobacteria (Betaproteobacteria)	YP_999693	Group-V *
Planctomyces limnophilus DSM 3776	Planctomycetes	YP_003630694	Group-IV *
Acidovorax avenae subsp. avenae ATCC 19860	Proteobacteria (Betaproteobacteria)	YP_004236344	Group-V *
Ralstonia eutropha JMP134	Proteobacteria (Betaproteobacteria)	YP_296790	Group-IV *
Clostridium perfringens D str. JGS1721	Firmicutes	ZP_02954019	Group-VIII *
Clostridium perfringens NCTC 8239	Firmicutes	ZP_02641467	Group-VIII *
Clostridium perfringens C str. JGS1495	Firmicutes	ZP_02865227	Group-VIII *
Clostridium perfringens B str. ATCC 3626	Firmicutes	ZP_02635767	Group-VIII *
Clostridium perfringens E str. JGS1987	Firmicutes	ZP_02631555	Group-VIII *
Clostridium perfringens CPE str. F4969	Firmicutes	ZP_02639308	Group-VIII *
Cupriavidus taiwanensis LMG 19424	Proteobacteria (Betaproteobacteria)	YP_002004803	Group-IV *
Achromobacter xylosoxidans A8	Proteobacteria (Betaproteobacteria)	YP_003977730	Group-V *
Polaromonas naphthalenivorans CJ2	Proteobacteria (Betaproteobacteria)	YP_981243	Group-V *
Clostridium kluyveri DSM 555	Firmicutes	YP_001397081	Group-VIII *
Clostridium kluyveri NBRC 12016	Firmicutes	YP_002473754	Group-VIII *
Acidovorax citrulli AAC00-1	Proteobacteria (Betaproteobacteria)	YP_972298	Group-V *
Clostridium beijerinckii NCIMB 8052	Firmicutes	YP_001307537	Group-VIII *
Achromobacter piechaudii ATCC 43553	Proteobacteria (Betaproteobacteria)	ZP_06686559	Group-IV *
Limnobacter sp. MED105	Proteobacteria (Betaproteobacteria)	ZP_01914658	Group-V *
Bordetella petrii DSM 12804	Proteobacteria (Betaproteobacteria)	YP_001630469	Group-IV *
Alicycliphilus denitrificans K601	Proteobacteria (Betaproteobacteria)	YP_004386669	Group-V *
Acidovorax ebreus TPSY	Proteobacteria (Betaproteobacteria)	YP_002552115	Group-V *
Acidovorax sp. JS42	Proteobacteria (Betaproteobacteria)	YP_984977	Group-V *
Comamonas testosteroni KF-1	Proteobacteria (Betaproteobacteria)	ZP_03545227	Group-V *
Comamonas testosteroni CNB-2	Proteobacteria (Betaproteobacteria)	YP_003276651	Group-V *
Comamonas testosteroni S44	Proteobacteria (Betaproteobacteria)	ZP_07043003	Group-V *
Clostridium botulinum B str. Eklund 17B	Firmicutes	YP_001884683	Group-VIII *
Achromobacter xylosoxidans A8	Proteobacteria (Betaproteobacteria)	YP_003977731	Group-IV *
Alicycliphilus denitrificans BC	Proteobacteria (Betaproteobacteria)	YP_004125434	Group-V *
Clostridium botulinum E1 str. 'BoNT E Beluga'	Firmicutes	ZP_04821422	Group-VIII *
Ralstonia sp. 5_7_47FAA	Proteobacteria (Betaproteobacteria)	ZP_07673817	Group-V *
Clostridium botulinum E3 str. Alaska E43	Firmicutes	YP_001919870	Group-VIII *
Ralstonia pickettii 12J	Proteobacteria (Betaproteobacteria)	YP_001892501	Group-V *
Ralstonia pickettii 12D	Proteobacteria (Betaproteobacteria)	YP_002984003	Group-V *
Bordetella petrii DSM 12804	Proteobacteria (Betaproteobacteria)	YP_001630468	Group-V *
Clostridium sp. 7_2_43FAA	Firmicutes	ZP_05132374	Group-VIII *

Lyngbya sp. PCC 8106	Cyanobacteria	ZP_01622608	Group-I *
Ralstonia sp. 5_7_47FAA	Proteobacteria (Betaproteobacteria)	ZP_07673818	Group-IV *
Ralstonia pickettii 12J	Proteobacteria (Betaproteobacteria)	YP_001892500	Group-IV *
Ralstonia pickettii 12D	Proteobacteria (Betaproteobacteria)	YP_002984002	Group-IV *
Herbaspirillum seropedicae SmR1	Proteobacteria (Betaproteobacteria)	YP_003775578	Group-IV *
Methylotenera sp. 301	Proteobacteria (Betaproteobacteria)	YP_003674412	Group-IV *
Gallionella capsiferriformans ES-2	Proteobacteria (Betaproteobacteria)	YP_003847387	Group-IV *
Bacillus sp. NRRL B-14911	Firmicutes	ZP_01172647	Group-XVIII
Polynucleobacter necessarius subsp. asymbioticus Q	Proteobacteria (Betaproteobacteria)	YP_001155453	Group-IV *
Candidatus Accumulibacter phosphatis clade IIA str	Proteobacteria (Betaproteobacteria)	YP_003169067	Group-IV *
Nitrosomonas eutropha C91	Proteobacteria (Betaproteobacteria)	YP_747614	Group-IV *
Herminiimonas arsenicoxydans	Proteobacteria (Betaproteobacteria)	YP_001101549	Group-IV *
Nitrosospira multiformis ATCC 25196	Proteobacteria (Betaproteobacteria)	YP_412935	Group-IV *
Acidovorax avenae subsp. avenae ATCC 19860	Proteobacteria (Betaproteobacteria)	YP_004236343	Group-IV *
Laribacter hongkongensis HLHK9	Proteobacteria (Betaproteobacteria)	YP_002795961	Group-IV *
Janthinobacterium sp. Marseille	Proteobacteria (Betaproteobacteria)	YP_001355245	Group-IV *
Nitrosomonas sp. AL212	Proteobacteria (Betaproteobacteria)	YP_004293564	Group-IV *
Acidovorax delafieldii 2AN	Proteobacteria (Betaproteobacteria)	ZP_04761261	Group-IV *
Acidovorax citrulli AAC00-1	Proteobacteria (Betaproteobacteria)	YP_972297	Group-IV *
delta proteobacterium MLMS-1	Proteobacteria (Deltaproteobacteria)	ZP_01291672	Group-XVII
Acidovorax ebreus TPSY	Proteobacteria (Betaproteobacteria)	YP_002552116	Group-IV *
Delftia acidovorans SPH-1	Proteobacteria (Betaproteobacteria)	YP_001566722	Group-IV *
delta proteobacterium MLMS-1	Proteobacteria (Deltaproteobacteria)	ZP_01288357	Group-XVII
Verminephrobacter eiseniae EF01-2	Proteobacteria (Betaproteobacteria)	YP_999694	Group-IV *
Acidovorax sp. JS42	Proteobacteria (Betaproteobacteria)	YP_984978	Group-IV *
Leptothrix cholodnii SP-6	Proteobacteria (Betaproteobacteria)	YP_001793058	Group-IV *
Comamonas testosteroni KF-1	Proteobacteria (Betaproteobacteria)	ZP_03545226	Group-IV *
Comamonas testosteroni S44	Proteobacteria (Betaproteobacteria)	ZP_07043004	Group-IV *
Comamonas testosteroni CNB-2	Proteobacteria (Betaproteobacteria)	YP_003276652	Group-IV *
Rhodoferax ferrireducens T118	Proteobacteria (Betaproteobacteria)	YP_522580	Group-IV *
Alicycliphilus denitrificans BC	Proteobacteria (Betaproteobacteria)	YP_004125435	Group-IV *
Alicycliphilus denitrificans K601	Proteobacteria (Betaproteobacteria)	YP_004386670	Group-IV *
Variovorax paradoxus EPS	Proteobacteria (Betaproteobacteria)	YP_004153457	Group-IV *
Variovorax paradoxus S110	Proteobacteria (Betaproteobacteria)	YP_002942976	Group-IV *
delta proteobacterium MLMS-1	Proteobacteria (Deltaproteobacteria)	ZP_01289304	Group-XVII
Oxalobacteraceae bacterium IMCC9480	Proteobacteria (Betaproteobacteria)	ZP_08273976	Group-IV *
Limnobacter sp. MED105	Proteobacteria (Betaproteobacteria)	ZP_01914659	Group-IV *
Polaromonas naphthalenivorans CJ2	Proteobacteria (Betaproteobacteria)	YP_981244	Group-IV *
Deinococcus deserti VCD115	Deinococcus-Thermus	YP_002787675	Group-XIX
Cyanothece sp. PCC 7822	Cyanobacteria	YP_003887952	Group-II *
Pelobacter propionicus DSM 2379	Proteobacteria (Deltaproteobacteria)	YP_901655	Group-XVII
Cyanothece sp. PCC 7424	Cyanobacteria	YP_002375941	Group-II *
Synechococcus sp. JA-2-3B'a(2-13)	Cyanobacteria	YP_479091	Group-II *
Oscillatoria sp. PCC 6506	Cyanobacteria	ZP_07109553	Group-II *
Cyanothece sp. CCY0110	Cyanobacteria	ZP_01731946	Group-II *
Thauera sp. MZ1T	Proteobacteria (Betaproteobacteria)	YP_002355630	Group-XVI
Nodularia spumigena CCY9414	Cyanobacteria	ZP_01628889	Group-II *
Cylindrospermopsis raciborskii CS-505	Cyanobacteria	ZP_06308120	Group-II *
Chlorobium phaeobacteroides BS1	Chlorobi	YP_001960471	Group-XVI
Kribbella flavida DSM 17836	Actinobacteria	YP_003381566	Group-X *
Congregibacter litoralis KT71	Proteobacteria (Gammaproteobacteria)	ZP_01104809	Group-VII *
Cyanothece sp. PCC 7425	Cyanobacteria	YP_002485117	Group-II *
gamma proteobacterium NOR5-3	Proteobacteria (Gammaproteobacteria)	ZP_05125958	Group-VII *
Clostridium perfringens D str. JGS1721	Firmicutes	ZP_02953772	Group-XVIII
Clostridium perfringens B str. ATCC 3626	Firmicutes	ZP_02636857	Group-XVIII
Clostridium perfringens CPE str. F4969	Firmicutes	ZP_02640360	Group-XVIII
Clostridium perfringens ATCC 13124	Firmicutes	YP_697134	Group-XVIII
Clostridium perfringens str. 13	Firmicutes	NP_563362	Group-XVIII
Clostridium perfringens NCTC 8239	Firmicutes	ZP_02643564	Group-XVIII
Clostridium perfringens E str. JGS1987	Firmicutes	ZP_02632845	Group-XVIII
Francisella novicida FTG	Proteobacteria (Gammaproteobacteria)	ZP_03247110	Group-VII *
Clostridium perfringens C str. JGS1495	Firmicutes	ZP_02865100	Group-XVIII
Francisella tularensis subsp. tularensis FSC033	Proteobacteria (Gammaproteobacteria)	ZP_04986698	Group-VII *
Francisella tularensis subsp. tularensis FSC198	Proteobacteria (Gammaproteobacteria)	YP_667234	Group-VII *
Francisella novicida GA99-3548	Proteobacteria (Gammaproteobacteria)	ZP_04990000	Group-VII *
Francisella novicida FTE	Proteobacteria (Gammaproteobacteria)	ZP_03057449	Group-VII *
Francisella tularensis subsp. tularensis MA00-2987	Proteobacteria (Gammaproteobacteria)	ZP_03665783	Group-VII *
Francisella tularensis subsp. tularensis WY96-3418	Proteobacteria (Gammaproteobacteria)	YP_001122089	Group-VII *
Francisella novicida U112	Proteobacteria (Gammaproteobacteria)	YP_898751	Group-VII *
Francisella tularensis subsp. tularensis MA00-2987	Proteobacteria (Gammaproteobacteria)	ZP_05247729	Group-VII *
Francisella novicida GA99-3549	Proteobacteria (Gammaproteobacteria)	ZP_04988556	Group-VII *
Francisella tularensis subsp. holarctica URFT1	Proteobacteria (Gammaproteobacteria)	ZP_06558954	Group-VII *
Francisella tularensis subsp. holarctica FSC200	Proteobacteria (Gammaproteobacteria)	ZP_02274704	Group-VII *
Francisella tularensis subsp. holarctica FSC022	Proteobacteria (Gammaproteobacteria)	ZP_04985202	Group-VII *
Francisella tularensis subsp. holarctica 257	Proteobacteria (Gammaproteobacteria)	ZP_04983546	Group-VII *
Francisella tularensis subsp. holarctica LVS	Proteobacteria (Gammaproteobacteria)	YP_513554	Group-VII *
Francisella tularensis subsp. holarctica FTNF002-0	Proteobacteria (Gammaproteobacteria)	YP_001428313	Group-VII *
Francisella tularensis subsp. holarctica OSU18	Proteobacteria (Gammaproteobacteria)	YP_763388	Group-VII *
Francisella philomiragia subsp. philomiragia ATCC	Proteobacteria (Gammaproteobacteria)	ZP_05249535	Group-VII *
Francisella philomiragia subsp. philomiragia ATCC	Proteobacteria (Gammaproteobacteria)	ZP_04755871	Group-VII *
Francisella tularensis subsp. mediasiatica FSC147	Proteobacteria (Gammaproteobacteria)	YP_001891928	Group-VII *
Francisella philomiragia subsp. philomiragia ATCC	Proteobacteria (Gammaproteobacteria)	YP_001678221	Group-VII *
gamma proteobacterium HdN1	Proteobacteria (Gammaproteobacteria)	YP_003810425	Group-XVI
Herpetosiphon aurantiacus ATCC 23779	Chloroflexi	YP_001546329	Group-VII *
Nakamurella multipartita DSM 44233	Actinobacteria	YP_003204092	Group-X *

Chlorobium chlorochromatii CaD3	Chlorobi	YP_379255	-
Campylobacterales bacterium GD 1	Proteobacteria (Epsilonproteobacteria)	ZP_05072003	Group-XVI
Caldicellulosiruptor lactoaceticus 6A	Firmicutes	ZP_07737651	Group-XIV
Caldicellulosiruptor hydrothermalis 108	Firmicutes	YP_003993205	Group-XIV
Caldicellulosiruptor saccharolyticus DSM 8903	Firmicutes	YP_001179563	Group-XIV
Caldicellulosiruptor bescii DSM 6725	Firmicutes	YP_002572426	Group-XIV
Caldicellulosiruptor kronotskyensis 2002	Firmicutes	YP_004024768	Group-XIV
Chlorobium chlorochromatii CaD3	Chlorobi	YP_379288	Group-XVI
Chlorobium chlorochromatii CaD3	Chlorobi	YP_379253	Group-XVI
Thauera sp. MZ1T	Proteobacteria (Betaproteobacteria)	YP_002355618	Group-XVI
Dethiobacter alkaliphilus AHT 1	Firmicutes	ZP_03729796	Group-III*
Dermacoccus sp. Ellin185	Actinobacteria	ZP_07705573	Group-X *
Kytococcus sedentarius DSM 20547	Actinobacteria	YP_003149146	Group-X *
Dethiobacter alkaliphilus AHT 1	Firmicutes	ZP_03729795	Group-III*
Bacillus halodurans C-125	Firmicutes	NP_244520	Group-III*
Bacillus halodurans C-125	Firmicutes	NP_244522	Group-III*
Thauera sp. MZ1T	Proteobacteria (Betaproteobacteria)	YP_002355619	Group-XVI
Plesiocystis pacifica SIR-1	Proteobacteria (Deltaproteobacteria)	ZP_01912972	Group-VII *
Oceanobacillus iheyensis HTE831	Firmicutes	NP_693841	Group-III*
Bacillus halodurans C-125	Firmicutes	NP_244521	Group-III*
Oceanobacillus iheyensis HTE831	Firmicutes	NP_693837	Group-III*
Caldicellulosiruptor kristjanssonii 177R1B	Firmicutes	YP_004026883	Group-XIV
Oceanobacillus iheyensis HTE831	Firmicutes	NP_693840	Group-III*

Groups which were originally described by Füser and Steinbüchel [1] are marked with an asterisk (*)

Reference

1. Füser G, Steinbüchel A (2007) Analysis of genome sequences for genes of cyanophycin metabolism: Identifying putative cyanophycin metabolizing prokaryotes. Macromol Biosci 7: 278-296.
