# Supplementary material for: Poles Apart: Arctic and Antarctic Octadecabacter strains Share High Genome Plasticity and a New Type of Xanthorhodopsin
Source: PLoS One. 2013 May 6;8(5):e63422. doi: 10.1371/journal.pone.0063422 (PMC3646047; doi:10.1371/journal.pone.0063422)
Supplement: Table S3 — List of metagenome projects used for the analyses of biogeography and diversity of microbial rhodopsins. (PDF) [file pone.0063422.s011.pdf]

Table S3. List of metagenome projects used for the analyses of biogeography and diversity of microbial rhodopsins.

Name/Description	Source	MG-RAST/CAMERA Accession	Sequencing Method	Reference
Antarctica aquatic microbial metagenome	CAMERA	CAM_PROJ_AntarcticaAquatic	454/ Sanger	[1]
Ward Hunt Ice Shelf melt pool metagenome	MG-RAST	WHI	454	[2]
Markham Ice Shelf melt pool metagenome	MG-RAST	MIS	454	[2]
Botany Bay metagenome	CAMERA	CAM_PROJ_BotanyBay	454/ Sanger	[3]
Global Ocean Sampling Expedition	CAMERA	CAM_PROJ_GOS	454/ Sanger	[4]
Ice metagenome of the northern Schneeferner	CAMERA	CAM_PROJ_IceMetagenome	454	[5]
Marine bacterioplankton metagenomes	CAMERA	CAM_PROJ_Bacterioplankton	454	[6]
Marine metagenome from coastal waters project at Plymouth Marine Laboratory	CAMERA	CAM_PROJ_PML	454	[7]
Metagenome from Yellowstone Bison Hot Spring	CAMERA	CAM_PROJ_Bison	454	[8]
Metagenomic analysis of the North Atlantic spring bloom	CAMERA	CAM_PROJ_BATS	454	-
Microbial community genomics at the HOT/ALOHA station	CAMERA	CAM_PROJ_HOT	Sanger	[9]
Monterey bay microbial study	CAMERA	CAM_PROJ_MontereyBay	454	[10]
Viral and microbial metagenomes from salterns of differing salinities	CAMERA	CAM_PROJ_SalternMetagenome	454	[11]
Yellowstone Lake: Genetic and gene diversity in a freshwater lake	CAMERA	CAM_PROJ_YLAKE	Sanger	[12]

References

1. Lauro F, DeMaere M, Yau S, Brown M, Ng C, et al. (2011) An integrative study of a meromictic lake ecosystem in Antarctica. ISME J 5: 879-895.
2. Varin T, Lovejoy C, Jungblut A, Vincent W, Corbeil J. (2010) Metagenomic profiling of Arctic microbial mat communities as nutrient scavenging and recycling systems. Limnol Oceanogr 55: 1901-1911.
3. Thomas T, Rusch D, DeMaere MZ, Yung PY, Lewis M, et al. (2010) Functional genomic signatures of sponge bacteria reveal unique and shared features of symbiosis. ISME J 4: 1557-1567.
4. Rusch D, Halpern A, Sutton G, Heidelberg K, Williamson S, et al. (2007) The Sorcerer II Global Ocean Sampling expedition: Northwest Atlantic through Eastern Tropical Pacific. Plos Biology 5: 398-431.
5. Simon C, Wiezer A, Strittmatter A, Daniel R. (2009) Phylogenetic Diversity and Metabolic Potential Revealed in a Glacier Ice Metagenome. Appl Environ Microbiol 75: 7519-7526.
6. Hewson I, Paerl RW, Tripp HJ, Zehr JP,Karl DM. (2009) Metagenomic potential of microbial assemblages in the surface waters of the central Pacific Ocean tracks variability in oceanic habitat. Limnol Oceanograph 54: 1981-1994.
7. Gilbert JA, Thomas S, Cooley NA, Kulakova A, Field D, et al. (2009) Potential for phosphonoacetate utilization by marine bacteria in temperate coastal waters. Environ Microbiol 11: 111-125.
8. Havig J, Raymond J, Meyer-Dombard D, Zolotova N, Shock E (2011) Merging isotopes and community genomics in a siliceous sinter-depositing hot spring. J Geophys Res 116: G01005.
9. Martinez A,Tyson GW, DeLong EF (2009) Widespread known and novel phosphonate utilization pathways in marine bacteria revealed by functional screening and metagenomic analyses. Environ Microbiol 12: 222-238.
10. Rich VI, Pham VD, Eppley J, Shi Y, DeLong EF (2011) Time-series analyses of Monterey Bay coastal microbial picoplankton using a 'genome proxy' microarray. Environ Microbiol 13: 116-134.
11. Dinsdale E, Edwards R, Hall D, Angly D, Breitbart M, et al. (2008) Functional metagenomic profiling of nine biomes. Nature 452: 629-632.
12. Kan J, Clingenpeel S, Macur R, Inskeep W, Lovalvo D, et al. (2011) Archaea in Yellowstone Lake. ISME J 5: 1784-1795.
