# Supplementary material for: Poles Apart: Arctic and Antarctic Octadecabacter strains Share High Genome Plasticity and a New Type of Xanthorhodopsin
Source: PLoS One. 2013 May 6;8(5):e63422. doi: 10.1371/journal.pone.0063422 (PMC3646047; doi:10.1371/journal.pone.0063422)
Supplement: Table S4 — Overview of selected features in regions of enhanced genome plasticity in the genomes of Octadecabacter arcticus (Oar-RGP1-17, pOAR118, pOAR160) and Octadecabacter antarcticus (Oan-RGP1-16, pOAN63). (PDF) [file pone.0063422.s012.pdf]

Table S4. Overview of selected features in regions of enhanced genome plasticity in the genomes of *Octadecabacter arcticus* (Oar-RGP1-17, pOAR118, pOAR160) and *Octadecabacter antarcticus* (Oan-RGP1-16, pOAN63).

Region	Protein-coding genes	Hypothetical genes	Transposable elements	Notable features
Oar-RGP1	12	3	9	-
Oar-RGP2	55	10	11	CO-dehydrogenase-like gene cluster; metabolism of aromatic compounds; amino-acid metabolism
Oar-RGP3	29	5	17	Fragment of flagella gene cluster; fragment of Type IV secretion system
Oar-RGP4	402	98	133	CO-dehydrogenase (OMP-type); fragment of gas vesicle gene cluster; thiamine metabolism; defect rhamnose uptake and utilization gene cluster; cyanase; diverse chaperons, unkown transport systems and various genes associated with carbohydrate and amino acid metabolism
Oar-RGP5	13	4	8	DNA-Methylase
Oar-RGP6	14	1	12	-
Oar-RGP7	25	5	17	Restriction endonuclease; exonuclease
Oar-RGP8	39	7	23	Ectoine uptake and utilization
Oar-RGP9	54	13	15	UV-induced DNA repair system; putative secondary metabolism; sulfurtransferase; diverse genes associated with fatty acid, carbohydrate and amino acid metabolism
Oar-RGP10	49	14	12	Glycin betain transporter; nitrile hydratase; resolvase; diverse genes associated with carbohydrate and amino acid metabolism
Oar-RGP11	97	26	32	Fragment of Type IV secretion system; topoisomerase; putative zinc transporter; UV-induced DNA repair system; diverse genes associated with carbohydrate and amino acid metabolism
Oar-RGP12	72	23	12	Thiamin metabolism; DNA-Helicase and exonuclease; riboflavin synthesis gene *ribH* ; diverse genes associated with carbohydrate, amino acid and energy metabolism
Oar-RGP13	100	20	35	Putative heavy metal efflux transporters; RNA-polymerase sigma factor; diverse genes associated with carbohydrate; amino acid and energy metabolism
Oar-RGP14	72	13	26	Mercury resistance; polysaccharide-synthesis; toxin/antitoxin plasmid stabilisation system; cholin/betain metabolism; helicase and topoisomerase
Oar-RGP15	68	22	36	Toxin/antitoxin plasmid stabilisation system; putative fimbria synthesis
Oar-RGP16	54	4	13	Multiple trimethylamine dehydrogenases; betain/cholin metabolism; diverse genes associated with amino acid and carbohydrate metabolism
Oar-RGP17	29	4	21	Xanthorhodopsin
pOAR118	122	51	22	Flagella synthesis; toxin/antitoxin plasmid stabilisation system; two different replication initiation proteins
pOAR160	141	22	30	Gas vesicle synthesis; toxin/antitoxin plasmid stabilisation system
Oan-RGP1	28	12	10	-
Oan-RGP2	26	10	3	Spermidin/putrescin transporter; RNA polymerase sigma factor
Oan-RGP3	68	21	5	CO-dehydrogenase (OMP-type); helicase; diverse genes associated with carbohydrate and nucleotide metabolism
Oan-RGP4	17	7	3	Cyanate hydratase
Oan-RGP5	37	19	3	Peptide transporter; putative secondary metabolism
Oan-RGP6	84	38	17	DMT- and RND-type efflux transporters
Oan-RGP7	20	12	2	-
Oan-RGP8	296	114	32	Rhamnose uptake and utilization; mercury resistance; DNA helicase, topoisomerase, recombinase, endonuclease and excisionase; cold shock protein; several defect UV-inducable DNA repair systems; diverse transporters of unkown function
Oan-RGP9	176	50	42	Flagella synthesis; gas vesicle synthesis; diverse genes associated with carbohydrate and amino acid metabolism
Oan-RGP10	64	27	8	Mercury resistance (defect); glycine/betaine transporter; diverse genes associated with amino acid metabolism
Oan-RGP11	22	13	7	Resolvase
Oan-RGP12	23	4	5	Hydantoin metabolism; diverse genes associated with amino acid and carbohydrate metabolism
Oan-RGP13	44	14	9	Sodium/hydrogen symporter; *tolQ* +*tolR* biopolymer transport proteins; sugar abc transporter
Oan-RGP14	10	5	3	-
Oan-RGP15	37	15	1	UV-inducable DNA repair system; iron + hemin uptake systems; recombinase/resolvase
Oan-RGP16	31	10	8	Xanthorhodopsin; DMT-type transporter; DNA helicase
pOAN63	57	10	5	Several transporters of unkown function, peptide transporters; toxin/antitoxin plasmid stabilisation system.
